# Post-transcriptional gene regulation by an Hfq-independent small RNA in *Caulobacter crescentus*

**DOI:** 10.1093/nar/gky765

**Published:** 2018-08-27

**Authors:** Kathrin S Fröhlich, Konrad U Förstner, Zemer Gitai

**Affiliations:** 1 Department of Molecular Biology, Princeton University, Lewis Thomas Laboratories, Princeton, NJ 08544, USA; 2 Department of Biology I, Microbiology, Ludwig-Maximilians-University Munich, D-82152 Martinsried, Germany; 3 Core Unit Systems Medicine, University of Würzburg, D-97080 Würzburg, Germany

## Abstract

Bacterial small RNAs (sRNAs) are a heterogeneous group of post-transcriptional regulators that often act at the heart of large networks. Hundreds of sRNAs have been discovered by genome-wide screens and most of these sRNAs exert their functions by base-pairing with target mRNAs. However, studies addressing the molecular roles of sRNAs have been largely confined to gamma-proteobacteria, such as *Escherichia coli*. Here we identify and characterize a novel sRNA, ChvR, from the alpha-proteobacterium *Caulobacter crescentus*. Transcription of *chvR* is controlled by the conserved two-component system ChvI-ChvG and it is expressed in response to DNA damage, low pH, and growth in minimal medium. Transient over-expression of ChvR in combination with genome-wide transcriptome profiling identified the mRNA of the TonB-dependent receptor ChvT as the sole target of ChvR. Genetic and biochemical analyses showed that ChvR represses ChvT at the post-transcriptional level through direct base-pairing. Fine-mapping of the ChvR-*chvT* interaction revealed the requirement of two distinct base-pairing sites for full target regulation. Finally, we show that ChvR-controlled repression of *chvT* is independent of the ubiquitous RNA-chaperone Hfq, and therefore distinct from previously reported mechanisms employed by prototypical bacterial sRNAs. These findings have implications for the mechanism and evolution of sRNA function across bacterial species.

## INTRODUCTION

The ability of bacteria to survive and grow in constantly changing conditions requires them to continuously adapt to their physical and chemical environments. The primary way in which bacteria adapt to different conditions is by altering their gene expression profiles, which they achieve by a complex interplay of transcriptional, post-transcriptional, translational, and post-synthetic processes. The most common molecular sensors in bacteria are two-component signal transduction systems (TCSs), which often span the bacterial inner membrane to translate external stimuli into intracellular, regulatory responses (1). Typically, bacterial TCSs consist of a membrane-inserted sensor kinase, which relays an external signal to a cognate cytoplasmic response regulator. When activated, most response regulators bind to genomic promoter elements, acting as transcriptional modulators (2). At the post-transcriptional level, bacteria frequently employ small, regulatory RNAs (sRNAs) to fine-tune gene expression. This versatile class of heterogeneously sized and structured RNA molecules predominantly acts by direct base-pairing to cognate target mRNAs, which typically requires the RNA chaperone, Hfq. Upon base-pairing, translation and/or stability of targeted transcripts are affected, leading to either repression or activation of gene expression (3).

Studies on the molecular functions of sRNAs have been strongly focused on enterobacterial organisms including *Escherichia coli* and *Salmonella* Typhimurium, and it has been suggested that every major regulon in these species contains at least one regulatory RNA (4). For example, ∼150–200 sRNAs have been discovered in *E. coli* to date, a small fraction of which has been characterized in more detail (5). In contrast, the roles of regulatory RNAs in the stalked alpha-proteobacterium *Caulobacter crescentus*, a well-established model of bacterial cell biology, have been barely addressed. Transcriptomic studies suggest that there are ∼140 sRNAs expressed from the *Caulobacter* genome during growth in rich and minimal medium (6). Most *C. crescentus* sRNAs remain uncharacterized with the exception of CrfA, an sRNA which is induced in response to carbon starvation and functions in remodeling the profile of outer membrane transport proteins under this condition (7), as well as GsrN, a conserved sRNA which is directly controlled by the general stress sigma factor, σ^T^, and facilitates expression of *katG* mRNA under hydrogen peroxide stress (8).

In many species, the activity of sRNAs depends on the ubiquitous RNA chaperone Hfq, which protects RNA from decay by ribonucleases and mediates base-pairing between sRNAs and cognate target transcripts (9). Given that *hfq* is an essential gene under certain growth conditions in *Caulobacter* (10), and that absence of Hfq is associated with a severe loss of fitness and an elongated cell morphology (11), Hfq-mediated sRNA activities are likely to also play a key role in this organism. Because post-transcriptional regulation represents a rapid mechanism for altering gene expression, harmful stress such as DNA damage, e.g. through UV-radiation, represents a potentially important context for studying sRNAs. Indeed, all bacteria have developed sophisticated stress response systems to ensure maintenance of genome integrity in the face of DNA damage. However, the *Caulobacter* DNA damage response differs significantly from the well-studied stress programs of enterobacteria such as *E. coli* (12–15). Consequently, here we set out to determine if and how regulatory RNAs are integrated into the *Caulobacter* response to DNA damage. Specifically, we performed a transcriptomic analysis of cells treated with the DNA-crosslinking agent mitomycin C (MMC). We identified one sRNA candidate, CCNA_R0100, which was induced by MMC, but also expressed during growth in minimal medium. Expression of CCNA_R0100 is controlled by the activity of the conserved ChvI-ChvG TCS, and we thus renamed the sRNA ChvR (ChvI-ChvG regulated RNA). ChvI-ChvG has been previously implicated in virulence regulation and the response to low pH in related alpha-proteobacteria, but our work represents its first implication in DNA damage and sRNA induction. We further discovered that ChvR acts as a negative regulator of the TonB-dependent receptor CCNA_03108/CC_3013 (hereafter: ChvT), and that ChvT production is repressed by ChvR under different physiological conditions. Mechanistically, ChvR employs two distinct base-pairing sites to interact with the *chvT* target transcript, and both sites are required for full regulation. Surprisingly, testing regulation in an *hfq* mutant strain revealed that both ChvR expression and ChvT repression occur independent of Hfq. In summary, our work presents the first in depth characterisation of an sRNA-target mRNA interaction in *C. crescentus*, and suggests that Hfq-independent processes could be an important aspect of post-transcriptional gene regulation in this organism.

## MATERIALS AND METHODS

### DNA oligonucleotides

Sequences of all oligonucleotides employed in this study are listed in Supplementary Table S2.

### Construction of plasmids

All plasmids used in this study are summarized in Supplementary Table S3.

Gibson assembly (16) was used to fuse flanking fragments of genes *vanAB* (f1: KFO-0065/KFO-0066, f2: KFO-0067/KFO-0068; pKF323-2), *chvR* (f1: KFO-0252/KFO-0253, f2: KFO-0254/KFO-0255; pKF379-1), *chvIG-hprK* (f1: KFO-0345/KFO-0346, f2: KFO-0347/KFO-0348; pKF389-6), and *chvT* (f1: KFO-0336/KFO-0338, f2: KFO-0337/KFO-0339; pKF436-1), with plasmid pNPTS138 at the multiple cloning site (KFO-0059/KFO-0060). The same approach was chosen to construct the ChvR transcriptional reporter pKF383-7; an *E. coli lacZ* fragment (amplified from pPR9TT using KFO-0286/KFO-0289) was fused with the *chvR* promoter region (spanning –109 to +9 relative to the transcriptional start; amplification by KFO-0282/KFO-0283 on *C. crescentus* gDNA) in backbone pXGFP-5 (PCR amplification by KFO-0277/KFO-0287).

To obtain pKF416-15, *3XFLAG::hfq* was amplified from KFS-0297 via KFO-0069/KFO-0072, restricted with MluI/BamHI and ligated to an equally treated pNPTS backbone.

The translational reporter plasmid pKF310-3 was constructed by inserting a XhoI/XbaI restricted fragment (spanning –480 to +30 of the *bapE* gene relative to the translational start; KFO-0029/KFO-0035 amplification of gDNA) into equally treated pKF308-1. pKF308 is a derivative of pPR9TT in which *lacZ* has been replaced by *sfgfp* (amplified from pXG10sf by KFO-0026/KFO-0028) via BamHI/SacI.

Plasmid pVan-ChvR (pKF382-1) was constructed by ligation of the *chvR* fragment (PCR-amplified from *C. crescentus* gDNA using KFO-0230/KFO-0231; the sense primer starts at the sRNA +1 site determined by transcriptome analysis and carries a 5′ phosphate modification; XbaI restriction) to the pBVMCS-6 backbone ((17); amplification with KFO-0056/KFO-0144 at the +1 site of the vanillate-inducible promoter; XbaI restriction).

A high-copy plasmid expressing ChvR under the control of its native promoter (pKF370-1; spanning region –96 relative to the TSS of *chvR* up to the 10^th^ aa of *recF*) was constructed by restricting pBXMCS-6 ((17); removal of xylose-responsive promoter) with PstI/XbaI, and ligation of an equally treated insert (PCR-amplified from *Caulobacter* gDNA using KFO-0227/KFO-0230).

For complementation of the *chvIG-hprK* deletion in the chromosome, the operon was amplified by PCR (KFO-0349/KFO-0350), and ligated to backbone pXGFP-5 ((17); integration into the *xyl* locus) via NheI/NdeI restriction sites (pKF390-14).

To express *gfp* reporter fusions under the control of P*rsaA*, plasmid pGFPC-2 (17) was PCR-amplified (KFO-0278/KFO-0321), restricted with NdeI and BglII, and ligated to an equally digested fragment spanning the *rsaA* upstream and promoter region to the transcriptional start site (amplified from gDNA using KFO-0323/KFO-0331). The resulting plasmid (pKF384-1) served as backbone to introduce different inserts amplified from gDNA at the at the second codon of *gfp* via cloning into EcoRI and KpnI restriction sites: pP*rsaA-rsaA::gfp* (pKF385-1; insert KFO-0483/KFO-0484); pP*rsaA-chvT::gfp* (pKF386-1; insert KFO-0326/KFO-0327); pP*rsaA-chvT-M1::gfp* (pKF397-4; insert KFO-0372/KFO-0327), pP*rsaA-chvT-del5::gfp* (pKF402-1; insert KFO-0382/KFO-0327).

Plasmids expressing single nucleotide mutants were constructed via PCR amplification of the original plasmids, DpnI digestion of template DNA, and self-ligation of purified PCR products. Plasmid pKF382-1 served as a template for PCR amplification with primer pairs KFO-0354/KFO-0355 (pVan-ChvR-M1; pKF395-1), KFO-0417/KFO-0418 (pVan-ChvR-M2; pKF414-1), and pKF395-1 was amplified using KFO-0417/KFO-0418 to obtain pVan-ChvR-M1M2 (pKF418-1). Correspondingly, plasmid pKF386-1 or pKF397-4 were amplified with primer pairs KFO-0426/KFO-0427 to obtain pP*rsaA-chvT-M2::gfp* (pKF420-1), and pP*rsaA-chvT-M1M2::gfp* (pKF466-1), respectively.

### Bacterial strains and growth conditions

A complete list of bacterial strains employed in this study is provided in Supplementary Table S4. The *Caulobacter crescentus* strain NA1000 (KFS-0006; lab stock Z. Gitai) is referred to as the wild-type strain and was used for mutant construction. Deletions and insertions in the *C. crescentus* chromosome were obtained by using a two-step recombination procedure (18). Chromosomal mutations were transferred by transduction with phage Cr30 following standard protocols.

*C. crescentus* was cultivated aerobically at 30°C in either complex PYE medium, or in minimal M2 salts containing 0.2% glucose (19). Where appropriate, media were supplemented with antibiotics at the following concentrations (liquid/solid): kanamycin (5/25 μg/ml); chloramphenicol (2/1 μg/ml); tetracycline (2/1 μg/ml); nalidixic acid (-/20 μg/ml). A final concentration of 0.5 mM vanillate was added to cultures to induce expression from the *vanAB* promoter. To induce DNA damage, bacteria were grown to mid-exponential phase (OD_660_ of 0.4) and treated with mitomycin C (1 μg/ml). The response to low pH was tested by growing cells in PYE (pH 7.0) to mid-exponential phase (OD_660_ of 0.5) when the cultures were split, collected by centrifugation and resuspended in fresh PYE medium (pH 7.0 or pH 5.5, respectively).

*E. coli* strains were grown aerobically at 37°C in LB broth. Where appropriate, medium was supplemented with antibiotics at the following concentrations: kanamycin (50 μg/ml); chloramphenicol (20 μg/ml); tetracycline (12 μg/ml); ampicillin (100 μg/ml).

### Transposon screen

For transposon mutagenesis, plasmid pRL27 ((20); carrying a hyperactive Tn5 transposase) was transferred to KFS-0172 by conjugation from an *E. coli* donor. Conjugants were selected on PYE agar supplemented with X-gal (40 μg/ml), kanamycin and nalidixic acid. White or light blue colonies were screened for integrity of the *lacZ* reporter, and Tn5 insertion sites were mapped as described previously (21).

### Protein sample analysis

To prepare whole-cell protein samples, bacteria were collected by centrifugation (3 min; 9 000 rpm; 4°C) and resuspended in 1× protein loading buffer (62.5 mM Tris–HCl, pH 6.8, 100 mM DTT, 10% (v/v) glycerol, 2% (w/v) SDS, 0.01% (w/v) bromophenol blue) to a final concentration of 0.01 OD/μl. To analyze protein levels by Western blotting, 0.1 OD per lane were separated by SDS-PAGE and transferred onto PVDF membranes. 3XFLAG-tagged fusion proteins were detected using anti-FLAG antibody (1:1 000; mouse; Sigma #F1804), respectively. DivJ served as a loading control, and was probed with an antiserum (1:1 000; rabbit; (22)).

### Fluorescence intensity measurements

Unless stated otherwise, GFP expression of translational reporter fusions was determined from cells cultivated overnight in PYE supplemented with the appropriate antibiotics and supplements. Samples were collected by centrifugation (3 min; 9 000 rpm; 4°C), washed once in phosphate buffer, and resuspended in phosphate buffer. A control sample not expressing GFP was used to determine background fluorescence. Fluorescence intensity in the presence of the control plasmid was set to 1, and relative expression was calculated from three biological replicates (error bars represent standard deviation).

### RNA isolation and Northern blot analysis

Total bacterial samples were collected, mixed with 0.2 volumes of stop-mix (95% ethanol and 5% phenol, v/v) and snap-frozen in liquid nitrogen. Total RNA was isolated using the Hot Phenol method with modifications (23). Pellets were resuspended in 600 μl lysozyme solution (0.5 mg/ml lysozyme in TE buffer, pH 8.0), and 60 μl of 10% (w/v) SDS. The suspension was incubated at 65°C in a water bath for 1–2 min. The pH was equilibrated by addition of 0.1 vol of sodium acetate (pH 5.2), and samples were mixed with 750 μl phenol. Tubes were incubated at 65°C for 5 min and frequently mixed. Samples were centrifuged (10 min; 13 000 rpm; 4°C), and the aqueous layer mixed with 750 μl chloroform and centrifuged again (10 min; 13 000 rpm; 4°C). The RNA was ethanol-precipitated from the aqueous layer, washed with 70% ethanol, dried and dissolved in water.

For Northern blot analysis, 5–10 μg of total RNA were separated on 6% polyacrylamide (7M urea) gels and electroblotted. Membranes were hybridized with gene-specific 5′ end-labelled DNA-oligonucleotides at 42°C in Roti-Hybri-Quick hybridization solution (Roth), and washed in three subsequent steps with SSC wash buffers (5×/1×/0.5× SSC) supplemented with 0.1% SDS.

### Hfq coIP

*C. crescentus* wild-type and cells expressing 3XFLAG-Hfq (KFS-0344) were grown in minimal M2G medium to OD_660_ of 1. Lysates of cell pellets corresponding to 50 OD_660_ were subjected to immunoprecipitation as described previously (24).

### Transcriptomic analysis using RNA-seq

Libraries for Illumina sequencing of cDNA were constructed by vertis Biotechnology AG, Germany (http://www.vertis-biotech.com/), as described previously for eukaryotic microRNAs (25) but omitting the RNA size-fractionation step prior to cDNA synthesis. For the depletion of processed transcripts, equal amounts of RNA were incubated with Terminator 5′-phosphate-dependent exonuclease (TEX; Epicentre) as previously described (26). The transcripts were not fragmented in order to get mainly sequencing reads of the 5′-end of the transcripts. In a second sample set, biological replicates were depleted from ribosomal RNA (Ribo-Zero rRNA Removal Kit (Bacteria); Epicentre) and fragmented using ultrasound (4 pulses of 30s each). Afterwards, RNAs <20 nt were removed using the Agencourt RNAClean XP kit (Beckman Coulter Genomics). Equal amounts of RNA samples were poly(A)-tailed using poly(A) polymerase. Then, the 5′-triphosphates were removed by applying tobacco acid pyrophosphatase (TAP) resulting in 5′-monophosphates. Afterwards, an RNA adapter was ligated to the 5′-phosphate of the RNA. First-strand cDNA was synthesized by an oligo(dT)-adapter primer and the M-MLV reverse transcriptase. In a PCR-based amplification step using a high fidelity DNA polymerase the cDNA concentration was increased to 20–30 ng/μl. A library-specific barcode for multiplex sequencing was part of a 3′-sequencing adapter. The resulting cDNA libraries were sequenced using an Illumina NextSeq HiSeq 2500 in single-end mode with 100 cycles for the unfragmented TEX treated and untreated sample, or an Illumina NextSeq 500 in single-end mode with 75 cycles for the fragmented libraries, respectively. The raw, de-multiplexed reads files have been deposited in the National Center for Biotechnology Information's Gene Expression Omnibus (GEO) (27), and are accessible via the GEO accession GSE104186 (http://www.ncbi.nlm.nih.gov/geo/query/acc.cgi?acc=GSE104186).

The Illumina reads in FASTQ format were trimmed with a cut-off phred score of 20 and cleaned from adapter sequences using cutadapt version 1.13. The following steps were performed using the subcommand ‘create’, ‘align’ and ‘coverage’ of the tool READemption (28) version 0.4.3. The poly(A)-tail sequences were removed and a size filtering step was applied in which sequences shorter than 12 nt were eliminated. The remaining reads were mapped to the reference genome sequences of *C. crescentus* NA1000 (accession number NC_011916.1, retrieved from NCBI Genbank) using segemehl (29,30). Based on the alignment files in BAM format, coverage files in wiggle format representing the number of aligned reads per base were created and visualized in the Integrated Genome Browser (30). Each graph was normalized to the total number of reads that could be mapped for the respective library. To restore the original data range each graph was then multiplied by the minimum number of mapped reads calculated over all libraries. For the differential gene expression analysis the number of aligned reads per genes were quantified and DESeq2 ((31); version 1.12.4) was applied to compare the two conditions. The data analysis workflow is compiled in a Unix Shell script that can be retrieved from Zenodo (https://doi.org/10.5281/zenodo.1028768).

### qRT PCR

To prepare cDNA samples for qRT PCR, RNA was extracted from two biological replicates using the SV Total RNA Isolation System (Promega), and reverse transcribed using SuperScriptIII (Invitrogen) following the manufacturers’ recommendations. Real-time PCR reactions were performed in 384-well optical reaction plates in technical triplicates on an ABI Prism 7900HT Sequence Detection System using with Sybr Green mix (Applied Biosystems). As an internal control, RNA abundances were normalized to *rpoD* mRNA levels.

### Microarray analysis

Total RNA samples were prepared from two independent biological replicates using the Hot Phenol method. Preparation of cDNA libraries, microarray hybridization (to customized Agilent microarrays, 0304061531; Agilent Technologies) and scanning were performed as described previously (32). Data analysis was performed using the Princeton University Microarray Database (PUMAdb), and microarray data were submitted to PUMAdb for archiving (https://puma.princeton.edu/cgi-bin/publication/viewPublication.pl?pub_no=580).

## RESULTS

### The *C. crescentus* transcriptome upon DNA damage

To analyze the involvement of sRNAs in the response to DNA damage in *Caulobacter*, we performed RNA sequencing before and after treatment with mitomycin C (MMC), a DNA-crosslinking drug and potent inducer of the DNA damage response. To confirm the induction of the stress response we used the previously characterized *Caulobacter*-specific endonuclease BapE as a reporter (12), and monitored expression of a transcriptional P*bapE::gfp* fusion in response to MMC treatment (Supplementary Figure S1). We isolated RNA from *Caulobacter crescentus* NA1000 grown to mid-exponential phase (OD_660_ of 0.4) in rich PYE medium, and after 240 min of growth in the presence of 1 μg/mL MMC (final OD_660_ of ∼1.0) when expression of the P*bapE::gfp* reporter had increased ∼5-fold (Supplementary Figure S1).

We used dRNA-seq (26) to quantify changes in transcript abundance under both conditions, and to define transcriptional start sites (TSS). The dRNA-seq protocol enriches primary transcripts by treating input RNA with terminator exonuclease, an enzyme selectively degrading mono-phosphorylated RNAs (as found on processed transcripts) while not affecting tri-phosphorylated RNAs (as found on primary transcripts).

**Figure 1.**
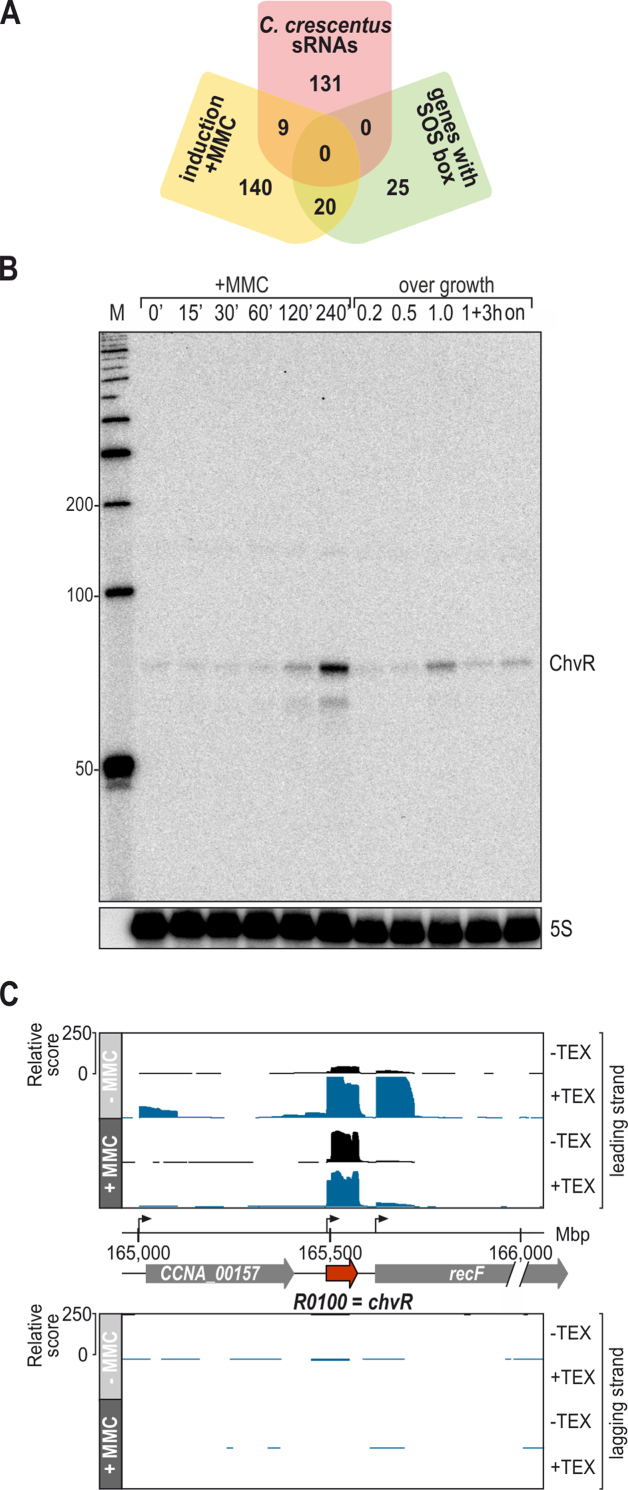
Transcriptome analysis of *C. crescentus* in response to DNA damage. (**A**) Diagram summarizing the results of the dRNA-seq experiment. RNA was collected from *C. crescentus* grown in PYE prior to (OD_660_ of 0.4) or 240 min after treatment with the DNA-damaging agent MMC. Under these conditions, 169 genes were induced >3-fold, including 9 sRNAs. No conserved SOS box was identified in proximity to transcriptional start sites of sRNAs. (**B**) Expression of ChvR sRNA in wild-type *C. crescentus*. Cells were grown in PYE, and RNA samples were collected prior to (OD_660_ of 0.4) and at indicated time-points after MMC treatment, or at different time-points over growth (OD_660_ of 0.2, 0.5, 1.0, 3h after cells had reached OD_660_ of 1.0, and overnight [on]), respectively. ChvR levels were determined by Northern blot analysis; 5S rRNA served as loading control. (**C**) cDNA reads of +/- MMC libraries mapping to the *chvR/recF* locus of *C. crescentus* NA1000. Libraries established from terminator exonuclease (TEX)-treated RNA samples are represented in blue, libraries established from untreated RNA in black, respectively. All libraries were adjusted to the same scale. Annotation and genome position are indicated in the centre. Transcriptional start sites are marked by arrows.

Mapping the cDNA reads to the *C. crescentus* NA1000 genome revealed differential expression (>3-fold induction) of 185 genes (Supplementary Table S1), including 9 transcripts annotated as sRNAs (Table 1; Figure 1A and Supplementary Figure S2). The master regulator of the SOS-response is LexA which, in the non-induced state, represses its target genes by binding to a regulatory motif, the so-called SOS box (13,33). Within the set of 185 genes induced in the presence of MMC, we identified 20 (out of 45 known) genes which have previously been identified to be controlled by LexA in *Caulobacter* (13). However, no SOS box (GTTCN_7_GTTC; (13,34)) was identified in proximity to the transcriptional start sites of MMC-induced sRNAs genes (Table 1).

**Table 1.** sRNAs upregulated in response to treatment with MMC

sRNA	Size (nt)	Own promoter	5′ flanking gene	3′ flanking gene	Orientation	Comment	Fold change	Specific induction by MMC
CCNA_R0158	95	yes	CCNA_02277	CCNA_02278	**> > >**		36.9	no
CCNA_R0097	84	no	CCNA_00028	CCNA_00027	**< < <**		18.3	no
CCNA_R0180	87	yes	CCNA_03118	CCNA_03117	**> < <**		4.9	no
CCNA_R0100	84	yes	CCNA_00157	CCNA_00158	**> > >**	ChvR	4.7	yes
CCNA_R0051	209	yes	CCNA_02253	CCNA_02254	**> > <**		4.4	no
CCNA_R0132	116	no	CCNA_01141	CCNA_01142	**> > >**	3′ UTR of CCNA_01141 (*recA*)	4.1	yes
CCNA_R0004	133	yes	CCNA_00197	CCNA_00196	**< < <**		3.7	no
CCNA_R0063	112	yes	CCNA_02725	CCNA_02726	**< > <**		3.3	no
CCNA_R0155	89	yes	CCNA_02158	CCNA_02158	**> > >**	internal TSS	3.1	no

*Caulobacter* can continue to grow in the presence of MMC such that genes induced by prolonged MMC exposure could represent genes induced by DNA damage or genes induced by changes in growth state such as entry into stationary phase. To distinguish these possibilities, we used Northern blot analysis to compare the RNA levels of our candidate sRNAs in the presence of MMC to the RNA levels in stationary phase (Supplementary Figure S3). Only two sRNAs, ChvR (CCNA_R0100) and CCNA_R0132, showed higher abundance in the presence of MMC compared to stationary phase growth in PYE (Figure 1B and Supplementary Figure S3E). Our dRNA-seq analysis revealed that sRNA CCNA_R0132 was not transcribed from its own promoter, but rather represented a stable RNA fragment derived from the 3′ UTR of the *recA* transcript (Supplementary Figure S2E). While such RNA species are in principle able to exert regulatory functions, they may also reflect accumulated RNA decay intermediates (24). We thus focused the remainder of our analysis on the sRNA ChvR, which is expressed under the control of its own promoter from the CCNA_00157/CCNA_00158 (*recF*) intergenic region as an 84 nt long transcript, and a less abundant processed RNA (Figure 1B, C and Supplementary Figure S4).

### Transcription of ChvR is controlled by the ChvI-ChvG TCS

**Figure 2.**
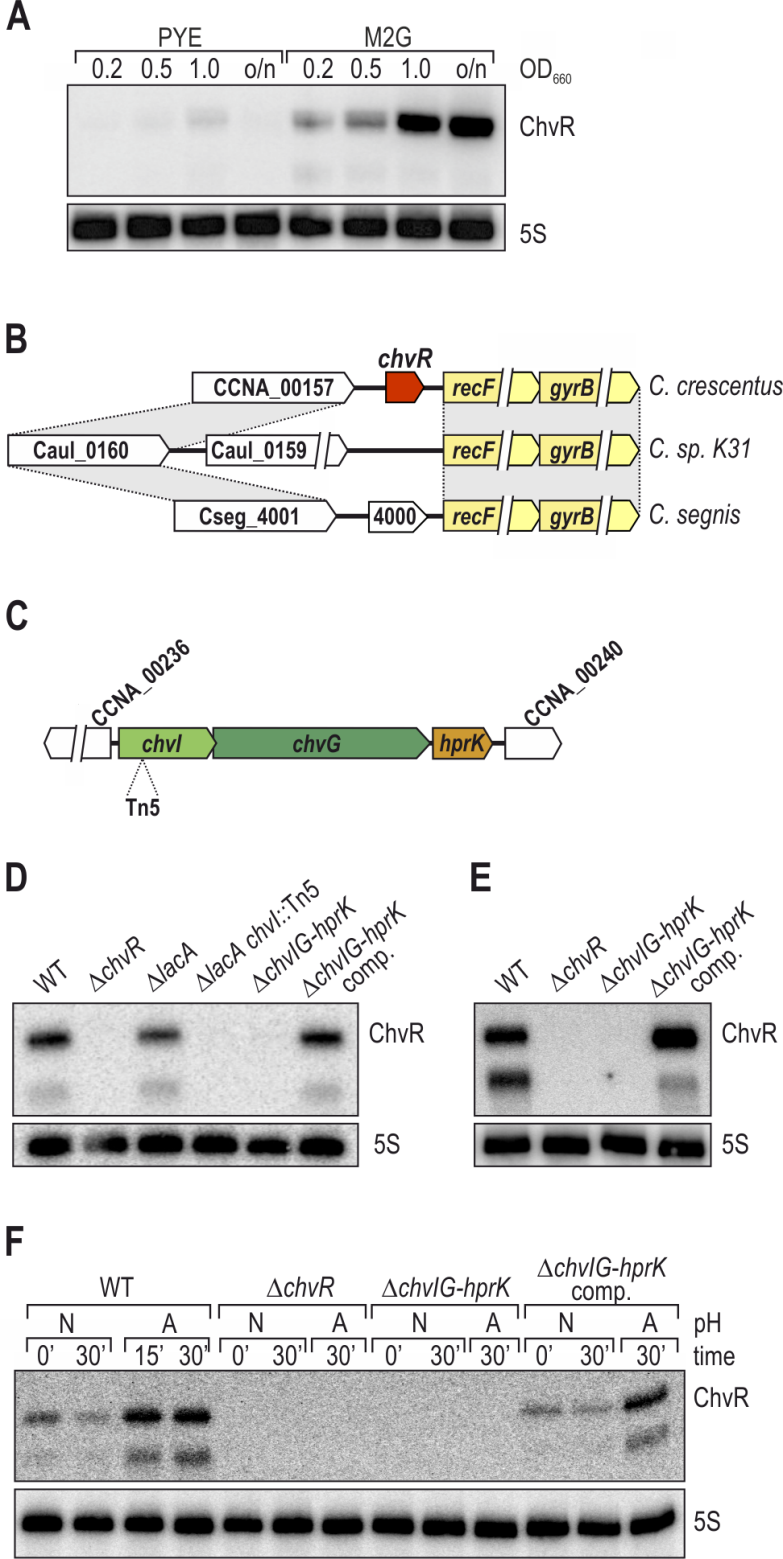
ChvR expression is controlled by the ChvI-ChvG TCS. (**A**) ChvR is induced in minimal medium. RNA was collected from *C. crescentus* grown in either PYE or M2G to indicated growth phases (OD_660_ of 0.2, 0.5, 1.0 and overnight [o/n]), and ChvR expression was determined by Northern blot analysis. (**B**) *chvR* is specific to *C. crescentus*. Synteny analysis of the genomic locus upstream the *recF/gyrB* operon between *C. crescentus, C. sp. K31* and *C. segnis*. Conserved regions are marked by gray boxes. (**C**) Mapping of the transposon insertion site. A transposon mutant with reduced P*chvR::lacZ* reporter activity was recovered, and the Tn5 insertion site was mapped to *chvI* (first gene of the *chvIG-hprK* operon). Flanking genes on the *C. crescentus* chromosome are indicated in white. (**D**) Expression of ChvR in minimal medium. ChvR expression was determined by Northern blot analysis of RNA samples collected from *C. crescentus* cultures grown in minimal M2G medium to mid-exponential phase. ChvR levels were compared between wild-type cells, the *chvR* deletion strain, the Δ*lacA* reporter strain, the Tn5 mutant recovered from the transposon screen, a chromosomal deletion of *chvIG-hprK*, and a complementation strain in which the *chvIG-hprK* operon is expressed under control of its own promoter from the *xyl* locus on the *C. crescentus* chromosome. (**E**) Expression of ChvR upon DNA damage requires integrity of the *chvIG-hprK* operon. RNA was collected from *C. crescentus* grown in PYE 240 min after treatment with MMC, and ChvR levels were determined by Northern blot analysis. (**F**) Expression of ChvR in acidified growth medium. *C. crescentus* was grown in PYE to mid-exponential phase when cultures were split, and growth was continued in PYE at either neutral (N; pH 7) or acidic pH (A; pH 5.5). RNA samples were collected at indicated time-points, and ChvR levels were determined by Northern blot analysis.

While ChvR is robustly induced from its own promoter upon DNA damage, we could not identify an SOS box in proximity to its transcriptional start site, indicating that it is controlled by a regulator other than LexA (Table 1). A tool for studying ChvR regulation emerged from a previous genomic analysis suggesting that ChvR becomes enriched during growth in minimal medium (6). To verify this result, we compared ChvR levels of *Caulobacter* grown in complex PYE or minimal M2G medium on Northern blots, and detected strong expression of the sRNA that increased during growth in minimal medium (Figure 2A). Gene synteny analysis revealed a high degree of plasticity in the region upstream of the *recF* gene (*i.e*. the genomic location of *chvR* in *C. crescentus*) in other *Caulobacter* species (Figure 2B), and BLAST searches (35) did not reveal conservation of the *chvR* gene beyond *C. crescentus*.

To determine the regulator responsible for ChvR induction we constructed a *lacZ* transcriptional reporter of *chvR*. In the presence of the chromogenic substrate X-gal, *Caulobacter* appears blue on solid media, and this native β-galactosidase activity is dependent on *lacA* (36). We thus chromosomally integrated our reporter into a *lacA* mutant background, in which the basal activity of X-gal hydrolysis is abolished. We randomly mutagenized this reporter strain using Tn5, and selected white or light blue colonies from PYE plates containing X-Gal. Tn5 insertions in the *lacZ* gene were excluded using PCR analysis. We isolated a single light-blue clone, and localized the transposon insertion site within *chvI*, interrupting the first gene of the *chvIG-hprK* operon (Figure 2C). We confirmed that the *chvI::Tn5* mutant disrupts ChvR induction in M2G by Northern blot analysis. When compared to wild-type, ChvR expression was not affected by the deletion of *lacA*, but dropped significantly in *chvI::Tn5* (Figure 2D; lanes 1, 3 and 4). Furthermore, ChvR expression was absent when the entire *chvIG-hprK* operon was deleted, but restored in a complementation strain in which the *chvIG-hprK* operon was expressed under control of its own promoter from the the *xyl* locus (lanes 5 and 6).

We next asked whether the *chvIG-hprK* operon was likewise required for induction of ChvR in response to DNA damage. To this end, we probed ChvR expression in RNA samples collected from MMC-treated cells and discovered that loss of the *chvIG-hprK* operon abolished MMC-dependent ChvR induction (Figure 2E). Thus, the *chvIG-hprK* operon is necessary for ChvR induction upon grown in both minimal media and MMC treatment.

Together, ChvG and ChvI form a TCS that is highly conserved in the alpha-proteobacteria (37–40), and ChvG has previously been shown to become activated in low pH environments (38). To test whether this was also the case in *Caulobacter*, we used ChvR induction as a proxy for ChvI-ChvG activity at neutral and acidic pH. Specifically, we cultivated wild-type *C. crescentus* in PYE medium to an OD_660_ of 0.5, pelleted the cells, and resuspended aliquots in either neutral or acidified PYE medium (pH of 7 or pH of 5.5, respectively). Total RNA was isolated prior to and after reinoculation, and ChvR levels were determined by Northern blot analysis. We discovered that ChvR expression was strongly induced during growth at low pH (Figure 2F, lanes 1–4), and that the *chvIG-hprK* locus was strictly required for ChvR induction (Figure 2F, lanes 8–10 versus 1–4 / 11–13). Taken together, our results suggest that ChvR sRNA expression is dependent on the ChvI-ChvG TCS, and that this TCS is largely inactive in rich PYE medium but active in minimal medium, at acidic pH, and during the DNA damage response of *C. crescentus*.

### ChvR is a trans-acting small RNA

**Figure 3.**
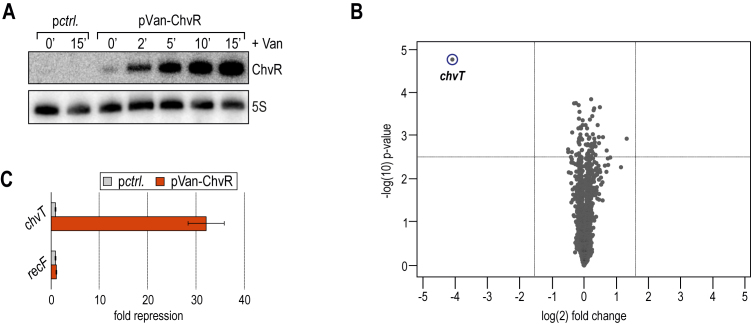
ChvR acts as a repressor of *chvT* mRNA. (**A**) *C. crescentus* Δ*chvR* cells carrying either plasmid pP*van*::ChvR (pKF382-1), or a control plasmid (pBVMCS-6) were grown in minimal M2G medium to mid-exponential phase (OD_660_ of 0.6) when vanillate was added to induce ChvR expression. RNA samples were collected prior to and at indicated time-points after sRNA induction, and ChvR levels were determined by Northern blot analysis. (**B**) Microarray analysis of *C. crescentus* genes affected by pulse overexpression of ChvR. Changes in transcript abundances between *C. crescentus* Δ*chvR* in response to ChvR overexpression and a control sample were scored on *C. crescentus*-specific microarrays. Dashed vertical or horizontal lines in the volcano plot indicate cut-off criteria of target selection (>3-fold change; *P*-value < 0.005), and *chvT* is marked in blue. (**C**) Verification of microarray results by qRT-PCR analysis. Abundances of *chvT* and *recF* mRNAs were determined in independent RNA samples collected as described in (**A**). The signal obtained in the control sample was set to 1; error bars represent the standard deviation calculated from two independent biological replicates.

To test if ChvR functions as a post-transcriptional regulator of gene expression in *C. crescentus*, we took a transcriptomic approach to screen for its direct targets. To minimize potential secondary effects of sRNA expression, *i.e*. the altered expression of an unrelated gene in response to deregulation of a direct target, we examined global mRNA changes in response to a brief pulse of ChvR overexpression (41,42). *Caulobacter chvR* mutant cells carrying either a plasmid expressing *chvR* under the control of the inducible *vanAB* promoter (pVan-ChvR), or an empty control vector were grown in M2G to an OD_660_ of 0.6. Since we had observed increased expression of ChvR in minimal medium, we suspected the sRNA to be active, and potential targets to be expressed under this condition. ChvR expression was induced by the addition of vanillate, and total RNA was collected prior to and at several time-points post induction. Northern blot analysis showed that ChvR was undetectable in the absence of vanillate, and that ChvR rapidly accumulated in the presence of the inducer (Figure 3A).

We scored changes in mRNA abundance on microarrays, and identified a single deregulated transcript (>3-fold) in response to ChvR over-expression (Figure 3B). The mRNA of CCNA_3108 (CC_3013; hereafter named *chvT*), encoding a TonB-dependent receptor, was repressed ∼15-fold in the presence of ChvR. To corroborate the transcriptome data, we also determined *chvT* levels using qRT-PCR. Consistent with our microarray results, the abundance of *chvT* mRNA was reduced ∼30-fold in the ChvR overexpression strain (Figure 3C). As a control, we also determined the effect of ChvR expression on *recF* mRNA, which is transcribed from the genomic locus just downstream of *chvR* in *Caulobacter* (Figure 3C, and Supplementary Figure S4). Our data showed that ChvR had no effect on the expression of the *recF* gene, indicating that ChvR acts *in trans* to specifically repress the *chvT* transcript.

### ChvR represses ChvT expression under various environmental conditions

**Figure 4.**
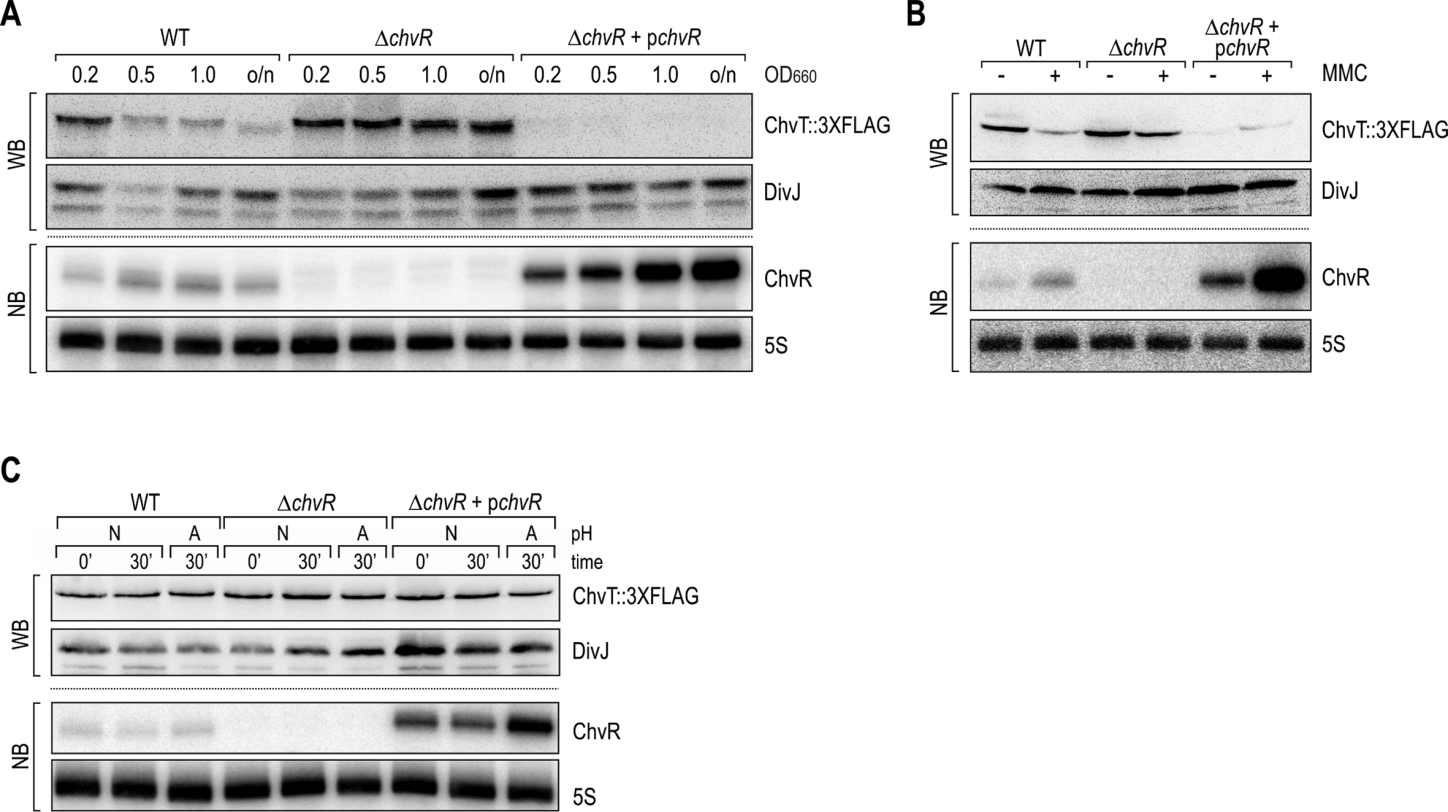
ChvR controls ChvT::3XFLAG production under different environmental conditions. (A–C) Samples were collected from *C. crescentus* wild-type (carrying a control plasmid (pBXMCS-6)), or a *chvR* deletion strain (carrying either a control plasmid, or a multi-copy construct expressing *chvR* from its own promoter (pKF370-1)). In all strains, *chvT* is marked by a C-terminal 3XFLAG affinity tag. Protein and RNA levels were determined by Western blot and Northern blot analysis, respectively. (**A**) *C. crescentus* cells were grown in minimal M2G medium to indicated growth phases (OD_660_ of 0.2, 0.5, 1.0 and overnight [o/n]). (**B**) Expression of ChvR and ChvT::3XFLAG was determined in *C. crescentus* grown in PYE prior to (OD_660_ of 0.5; [-]), and 240 min after treatment with MMC [+]. (**C**) *C. crescentus* was grown in PYE to mid-exponential phase (OD_660_ of 0.5) when cultures were split, and growth was continued in PYE at either neutral (N; pH 7) or acidic pH (A; pH 5.5).

To further characterize ChvR-mediated control of *chvT*, we investigated whether the repression we identified from an overexpression pulse also occurred under physiological conditions when ChvR was expressed from its endogenous promoter. To this end, we added a C-terminal 3XFLAG affinity tag to the chromosomal locus of *chvT*, and monitored production of ChvT::3XFLAG in wild-type *C. crescentus* as well as Δ*chvR* mutant cells carrying either an empty control vector or a high-copy plasmid complementing *chvR* under control of its native promoter. We first compared the levels of ChvT::3XFLAG from cells grown in M2G and observed increased expression of ChvT in the absence of ChvR in all growth phases (Figure 4A, lanes 1–4 and 5–8). ChvT::3XFLAG became undetectable in cells in which the ChvR sRNA deletion was complemented by a high-copy plasmid expressing *chvR* (Figure 4A, lanes 9–12). As expected, the ChvT::3XFLAG protein pattern was anti-correlated with the expression of the ChvR sRNA (Figure 4A, lower panel).

ChvR sRNA was also induced in *Caulobacter* cells treated with MMC (Figures 1B and 4B), and expression of ChvT::3XFLAG decreased in response to DNA damage (Figure 4B, lanes 1 and 2). In contrast, ChvT levels remained constantly high in a *chvR* mutant irrespective of the addition of MMC (lanes 3 and 4). Overexpression of the sRNA from the complementation plasmid reduced ChvT::3XFLAG both prior to and after addition of MMC to the culture (lanes 5 and 6).

In contrast to our findings with minimal media and MMC treatment, monitoring ChvT::3XFLAG expression revealed no detectable changes in protein levels, independent of ChvR expression within 30 min of growth at low pH (Figure 4C). Bacteria are generally able to adapt rapidly to mildly acidic conditions (43). Indeed, monitoring ChvR expression during extended growth at low pH for up to 120 min revealed that the sRNA was only transiently induced with a peak in expression at 30 min after shifting the cultures (Supplementary Figure S5). Given that membrane proteins are usually highly stable (44), and that the growth rate of *C. crescentus* is reduced under acidic conditions, transient induction of ChvR appears to be insufficient to significantly change ChvT protein levels.

### ChvR base-pairs with *chvT* mRNA via two distinct base-pairing sites

**Figure 5.**
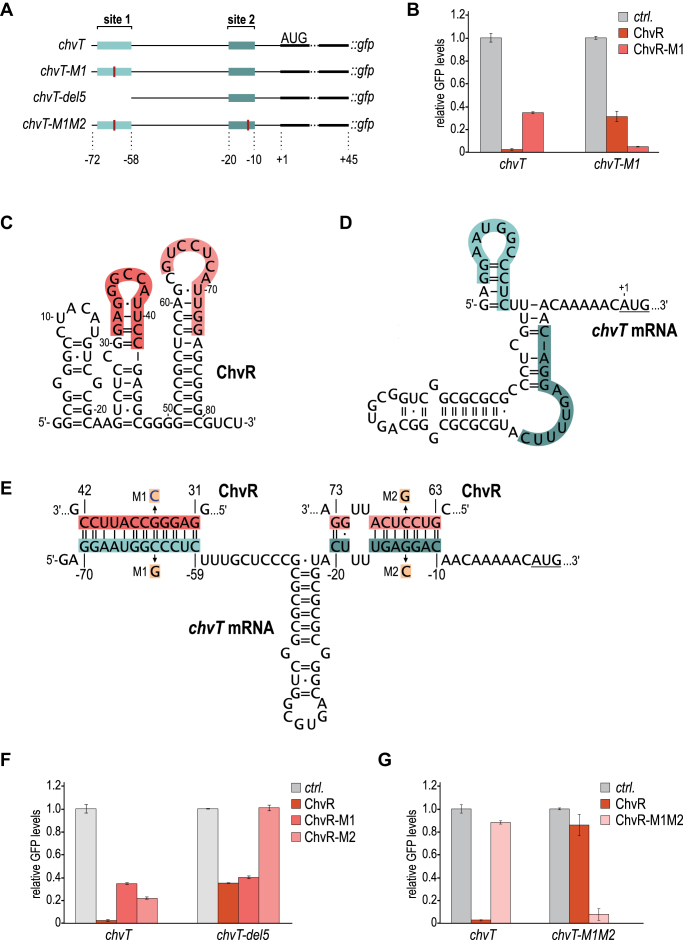
ChvR interacts with two independent sites of *chvT* mRNA. (**A**) Schematic representation of the translational *chvT::gfp* fusions (under control of the constitutive P*rsaA* promoter). Reporters comprise the 5′ untranslated region plus the first 45 nucleotides of the *chvT* CDS. Location of ChvR binding sites 1 and 2 are indicated by blue boxes, positions of single nucleotide exchanges are marked in red. (**B**) Partial repression of *chvT::gfp* by ChvR via binding site 1. *C. crescentus* Δ*vanAB* carrying the indicated *gfp* reporter fusion were co-transformed with either a control plasmid (pBVMCS6), or a plasmid overexpressing ChvR or ChvR-M1 under the control of the vanillate-inducible promoter P*van* (pKF382-1 or pKF395-1, respectively) were grown overnight in PYE supplemented with vanillate. GFP fluorescence was determined by plate reader measurements. For each fusion, GFP levels in the presence of the control plasmid were set to 1, and relative changes were determined for cells expressing ChvR. GFP levels were calculated from three biological replicates; error bars indicate the standard deviation. (**C**, **D**) Secondary structures of ChvR and the 5′ UTR of *chvT* mRNA (from the transcriptional start site to the start codon) based on bioinformatic predictions (45). (**E**) Predicted base-pairing interactions forming between ChvR and *chvT* mRNA. Both interactions at binding site 1 (base-pairing of the second stem-loop of ChvR (nts 31–42) and *chvT* mRNA (nts −70 to −59 relative to the translational start site)) and at binding site 2 (base-pairing of the third stem-loop of ChvR (nts 63–73) and *chvT* mRNA (nts −20 to −10 relative to the translational start site). Positions of single-nucleotide exchanges generating the compensatory mutants M1 and M2 are indicated. Expression of all ChvR variants was confirmed by Northern blot analysis (Supplementary Figure S7B). (**F-G**) Analysis of GFP fluorescence of *C. crescentus* Δ*vanAB* carrying the indicated *gfp* reporter fusion in combination with either a control construct, or plasmids overexpressing ChvR, ChvR-M1, ChvR-M2 (pKF414-1) or ChvR-M1M2 (pKF418-1). Experimental details as in (B).

How does ChvR regulate *chvT?* Bacterial sRNAs frequently function at the post-transcriptional level by base-pairing with the 5′ region of target mRNAs, altering transcript stability and translation (3). To uncouple the expression of *chvT* mRNA from its endogenous transcriptional control, we constructed a post-transcriptional reporter expressing the 5′ UTR and the first 15 codons of *chvT* fused to the green fluorescent protein (GFP) and drove transcription of this reporter with the constitutive P*rsaA* promoter (Figure 5A). As validated by qRT-PCR, overexpression of ChvR did not influence *rsaA* expression levels (Supplementary Figure S6). We integrated our reporter construct at the native *rsaA* locus, and co-transformed cells with either plasmid-borne pVan-ChvR or an empty control vector. Analyzing GFP production from this reporter fusion in cells grown for 12 h in the presence of vanillate revealed strong (∼15-fold) repression of GFP (Figure 5B), confirming that ChvR regulates *chvT* at the post-transcriptional level.

Bioinformatic predictions (using RNAfold; (45)) indicated that ChvR is likely highly structured, forming three hairpins with the last hairpin potentially acting as a rho-independent transcription terminator (Figure 5C). To investigate how ChvR regulates the expression of *chvT*, we used the RNAhybrid algorithm to predict base-pairing between the sRNA and the target transcript (46). This analysis predicted the formation of a continuous 12 bp interaction between the very 5′ end of the *chvT* mRNA and the second stemloop of ChvR (Figure 5C–E). To validate this prediction, we introduced a point mutation at position 35 in ChvR, replacing a guanosine with a cytosine (ChvR-M1; Figure 5E). While this mutation strongly reduced the repression of the reporter, ChvR-M1 still significantly down-regulated ChvT::GFP levels when compared to the control sample (∼5-fold; Figure 5B). Regulation of the *chvT* reporter was specific as expression of a control reporter P*rsaA::gfp* was only mildly affected by the presence of ChvR (Supplementary Figure S7A). We next introduced a compensatory mutation in the *chvT::gfp* reporter (*chvT-M1*, replacing a guanosine at position –62 relative to the translational start site with a cytosine residue; Figure 5E) and observed that wild-type ChvR had an intermediate effect (∼3-fold repression) on this reporter, whereas co-expression of ChvR-M1 fully restored regulation (Figure 5B). These results suggest that full repression of *chvT::gfp* by ChvR depends on the formation of a second base-pairing site between the two RNAs.

To identify candidates for the second interaction site we first shortened the *chvT::gfp* reporter from the 5′ end to eliminate the primary binding site (*chvT-del5::gfp*; Figure 5A). Both ChvR and ChvR-M1 repressed *chvT-del5::gfp* to a similar extent (∼3-fold; Figure 5F), confirming our hypothesis that ChvR uses an additional base-pairing site to repress this reporter. Based on this information, we predicted an alternative interaction between the third stem–loop of ChvR and a region just upstream of the translational start site of *chvT* mRNA (Figure 5D and E). A single-nucleotide exchange in ChvR (ChvR-M2; replacing cytosine at position –13 with a guanosine; Figure 5E) partially reduced GFP levels of the *chvT* wild-type reporter and fully abrogated repression of *chvT-del5::gfp* (Figure 5F).

We confirmed the requirement of both interaction sites by introducing two individual single nucleotide exchanges in ChvR. The sRNA variant ChvR-M1M2 completely failed to repress the wild-type *chvT::gfp* reporter (Figure 5G). Likewise, a *chvT-M1M2::gfp* reporter was no longer regulated by wild-type ChvR, but was strongly repressed by ChvR-M1M2. Together, our data shows that ChvR controls *chvT* at the post-transcriptional level, and that two distinct base-pairing sites are required for full regulation.

### ChvR-mediated regulation is independent of the chaperone Hfq

**Figure 6.**
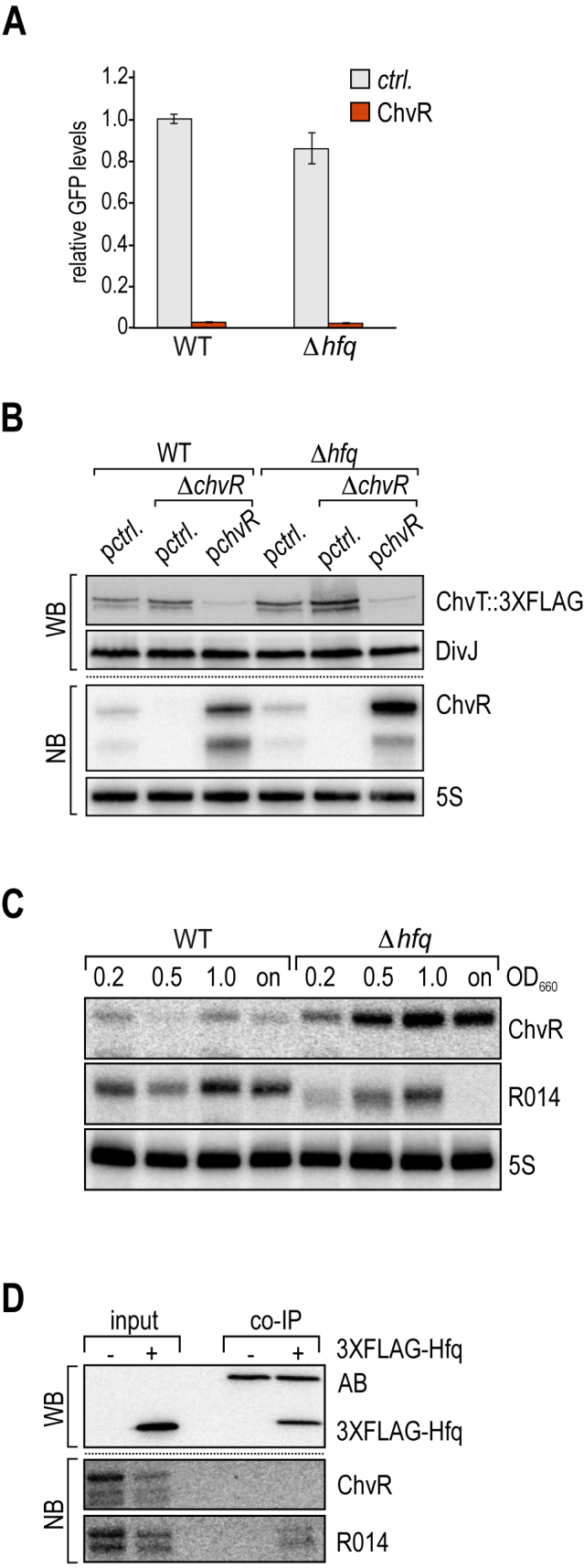
Post-transcriptional regulation by ChvR is independent of Hfq. (**A**) Analysis of GFP fluorescence of *C. crescentus* Δ*vanAB* (WT) or Δ*vanAB* Δ*hfq* (Δ*hfq*) cells carrying the indicated *gfp* reporter fusion in combination with either a control construct, or a plasmid overexpressing ChvR. Experimental details as in Figure 5 (B). (**B**) Samples were collected from *C. crescentus* wild-type (carrying a control plasmid (pBXMCS-6)), or a *chvR* deletion strain (carrying either a control plasmid, or a multi-copy construct expressing *chvR* from its own promoter (pKF370-1)), as well as isogenic *hfq* mutants of these strains grown in minimal M2G medium to an OD_660_ of 0.5. In all strains, ChvT is marked by a C-terminal 3XFLAG affinity tag. Protein and RNA levels were determined by Western blot and Northern blot analysis, respectively. (**C**) Expression of ChvR in an *hfq* mutant strain. *C. crescentus* wild-type and Δ*hfq* cells were grown in M2G to indicated time-points over growth (OD_660_ of 0.2, 0.5, 1.0 and overnight [on]). RNA expression of ChvR and CCNA_R0014 were determined by Northern blot analysis. (**D**) ChvR does not interact with Hfq *in vivo. C. crescentus* wild-type or a *3XFLAG::hfq* strain were grown in minimal M2G medium to OD_660_ of 1.0, and cell lysates were subjected to immunoprecipitation using a monoclonal anti-FLAG antibody. Protein and RNA samples of lysate (input) and co-immunoprecipitation fractions (co-IP) were analysed by Western and Northern blot analysis, respectively. AB: recovered anti-FLAG antibody.

In most well-characterized bacteria the formation of intermolecular base-pairing between cognate sRNA/mRNA partners is aided by the RNA chaperone, Hfq (9). Due to a role in the maintenance of central metabolism, deletion of *hfq* is associated with severe phenotypes including loss of cell morphology in *C. crescentus* (11), and is even an essential gene under certain growth conditions (10). To investigate the potential involvement of Hfq in regulating *chvT* mRNA by ChvR, we tested regulation of the *chvT::gfp* reporter in a *C. crescentus hfq* mutant strain. We determined that absence of the chaperone mildly reduced basal expression levels of *chvT::gfp*, but did not affect repression by ChvR (Figure 6A). It has previously been reported that certain Hfq-dependent sRNAs also exert regulatory activity in the absence of the chaperone when strongly overexpressed (47). To verify our observation that ChvR functions without Hfq, we compared the synthesis of ChvT::3XFLAG in wild-type *C. crescentus* and Δ*chvR* mutant cells (carrying either an empty control vector or a high-copy plasmid complementing *chvR* under its native promoter) to isogenic Δ*hfq* or Δ*hfq* Δ*chvR* mutant strains grown in minimal M2G medium (Figure 6B). Similar to the expression pattern of wild-type *C. crescentus*, ChvT::3XFLAG was more abundant in the *hfq* mutant strain in the absence of ChvR, and barely detectable in cells in which Δ*chvR* was complemented. We also observed that ChvR expression was increased in the *hfq* mutant (Figure 6B, lower panel). We next examined the abundance of ChvR in the *hfq* mutant strain in comparison to wild-type cells at different time-points over growth. In further support of the Hfq-independent phenotype observed before, Northern blot analysis revealed that expression of ChvR was indeed not reduced in the absence of Hfq, but that the sRNA was present at even higher levels when compared to the wild-type strain (Figure 6C).

Since we did not observe increased levels of ChvR in *C. crescentus* Δ*hfq* when expressing the sRNA from the vanillate-inducible promoter in our reporter assays (Supplementary Figure S9) we speculate that deletion of *hfq* results in activation of the *chvR* promoter.

Since the independence of ChvR function and expression from Hfq was surprising, we sought an additional method to investigate ChvR’s relation to Hfq. We thus asked if ChvR biochemically interacts with *Caulobacter* Hfq. To this end, we subjected wild-type and *3XFLAG::hfq C. crescentus* lysates to immunoprecipitation with a monoclonal anti-FLAG antibody and compared co-purified RNA to total RNA samples of both strains. When probing for ChvR, we found the sRNA to be absent from co-IP fractions but detected a signal in total RNA samples (Figure 6D). We furthermore probed for sRNA CCNA_R0014, which is a homologue of AbcR1, an sRNA associated with Hfq in the distantly related alpha-proteobacterium *Sinorhizobium meliloti* (48). We detected CCNA_R0014 in both samples of total RNA, but also specifically enriched in the Hfq co-IP fraction. Moreover, expression of R0014 was reduced in the *hfq* mutant strain compared to wild-type *C. crescentus* (Figure 6C). This result suggests that only a subset of sRNAs in *Caulobacter* associate with the RNA-binding protein Hfq, and that *Caulobacter* has a second, Hfq-independent class of sRNAs that includes the sRNA ChvR. While *C. crescentus* does not encode homologues of the two other global sRNA binding protein families in bacteria, CsrA/RsmA and ProQ, respectively, we cannot exclude that ChvR requires a different, yet-to-be-identified accessory factor to inhibit *chvT* expression.

## DISCUSSION

Gene regulation by sRNAs plays an important role in the physiology of many microorganisms (49). The impact of bacterial sRNAs on post-transcriptional regulation has been intensively studied in the gamma-proteobacteria *E. coli* and *Salmonella*, but the biological function of the majority of sRNAs identified to date is unknown. In this work, we characterized a novel sRNA, ChvR, in *C. crescentus* which is expressed under the control of the conserved ChvI-ChvG TCS in response to DNA damage, acidic pH and growth in minimal medium. ChvR functions as a dedicated repressor of the TonB-dependent receptor, ChvT. Acting as a post-transcriptional regulator, ChvR recognizes *chvT* mRNA at two distinct sites, and both base-pairing interactions are required for full repression. Importantly, the expression and regulatory activity of ChvR are independent of the conserved RNA chaperone, Hfq. Our results suggest that while *Caulobacter* Hfq does associate with some sRNAs, ChvR represents a class of Hfq-independent sRNAs that contribute to the adjustment of gene expression in this species.

### ChvR represses the TonB-dependent receptor ChvT

ChvT is one of 65 TonB-dependent receptor proteins in *C. crescentus* (50) and has a possible function for the survival of *C. crescentus* under nutrient scarce conditions (51). TonB-dependent receptors can facilitate the transport of siderophores, vitamins or carbohydrates (52), but as for most of the members of this class, the substrate of ChvT is not known. In general, bacterial outer membrane proteins serve as selective barriers controlling the exchange of both harmful and beneficial substances, and thus their expression is usually tightly regulated (53). One important layer of control of outer membrane protein expression is constituted by sRNAs, which have been repeatedly identified to regulate membrane composition and architecture at the post-transcriptional level (54). Indeed, another characterized sRNA from *C. crescentus*, CrfA, also modulates the expression of a large set of outer membrane proteins (7). It is currently not fully understood why mRNAs translating into outer membrane proteins could be more prone to regulation by sRNAs than other transcripts. One possible explanation is the relatively long half-live paired with the high copy number of some outer membrane protein-coding transcripts, and the potential need to rapidly reduce transcript numbers when environmental conditions change. Under these circumstances, efficient sRNA-mediated regulation could be superior to conventional transcriptional control mechanism of outer membrane protein synthesis.

### ChvR expression is controlled by the ChvI-ChvG TCS

*Caulobacter* thrives in nutrient-poor aquatic habitats (55), a lifestyle which requires the bacterium to cope with a diverse range of physiological stresses. The ability to adapt to ever changing conditions is reflected in the high number of two-component systems (34 sensor kinases, 44 response regulators and 27 sensor kinase/response regulator hybrid genes) encoded in the *C. crescentus* genome (50), which function in the transmission of signals to regulate response processes.

The ChvI-ChvG TCS is highly conserved among the alpha-proteobacteria, and is required for the association of bacteria with higher organisms. For example, mutations in either *chvI* or *chvG* abrogate the ability of *Agrobacterium tumefaciens* to form tumors on plants (56), and mutants of the homologous BvrR-BvrS TCS in *Brucella abortus* display reduced virulence in animal and cell culture models (40). The ChvI-ExoS TCS of *S. meliloti* is crucial for the establishment of symbiosis between the bacterium and its host plant alfalfa, and regulating the production of succinoglycan (37).

In this study, we showed that expression of the ChvR sRNA in *C. crescentus* is controlled by the ChvI-ChvG TCS (Figure 2D), and that ChvR is produced in response to DNA damage, acidic pH and during growth in minimal medium (Figure 2D–F). Since *C. crescentus* is a free-living, non-pathogenic bacterium, our results suggest an additional role of the ChvI-ChvG TCS other than sensing a host cell environment. The environmental cues perceived by membrane receptors are oftentimes unknown (40,57), and this is also the case for ChvG. Whether one common or several different cues activate signaling through the ChvI-ChvG TCS in *Caulobacter* under the different inducing conditions remains to be determined.

### Requirement of two individual binding sites

Post-transcriptional regulation of *chvT* mRNA by ChvR is based on the formation of two distinct RNA-RNA interactions between the sRNA and its target (Figure 5E). While full repression requires ChvR to bind both target sites on the *chvT* mRNA (Figure 5G), each individual base-pairing confers an intermediate effect on target gene expression (Figure 5B, F). Given that ChvR uses two different sequence stretches to base-pair at the very 5′ end of the *chvT* transcript or close to the ribosome binding site, respectively, it remains to be established whether one or two sRNA molecules are required to form these interactions. Multi-site pairing of an sRNA on its mRNA target is not common, but has been observed previously. For example, the *E. coli* sRNA Spot42 employs two different seed regions to repress *nanC, galK, sthA* and *ascF* mRNAs. OxyS sRNA, involved in the *E. coli* response to oxidative damage, forms two kissing loop complexes with the *fhlA* transcript, and repression of *lpxR* mRNA by MicF likewise involves two distinct base-pairing interactions between the binding partners. Unlike ChvR, however, these sRNAs require Hfq to function (58–60). Structurally, Hfq can only accommodate one sRNA-mRNA pair at a time (61). Thus, the activity of the *E. coli* sRNAs may be restricted from targeting both mRNA sites simultaneously. In contrast, the Hfq-independent two-site pairing discovered here for ChvR (Figure 6) could provide a novel mechanism for using two sites simultaneously to enhance both the strength and efficiency of target regulation by the sRNA.

The locations of the two pairing sites within its mRNA target suggest that the two base-pairing regions of ChvR regulate *chvT* expression via distinct mechanisms. The base-pairing between ChvR and *chvT* at site 2 is formed by 7 + 2 bp interrupted by a 2 bp bulge (–15.5 kcal/mol (46); Figure 5E). By binding close to the start codon (residues –10 to –20 relative to the AUG of *chvT* mRNA), ChvR could mask the recognition site for the 30S ribosomal subunit (ranging from residue -20 in the 5′ UTR to +19 in the coding sequence; (62)) and thereby interfere with translation initiation (63). In contrast, base-pairing site 1 is located at the very 5′ end of the *chvT* mRNA at residues –70 to –59 relative to the AUG (–27.6 kcal/mol (46); Figure 5E), and thus is in considerable distance from translation initiation signals. Bioinformatic predictions of the secondary structure of the *chvT* leader (using RNAfold; (45)) reveal the formation of a weak stem-loop structure that would be interrupted by interacting with ChvR (see Figure 5D and E). Similar structural elements have previously been shown to confer stability to transcripts by occluding the access of exonucleases with 5′ to 3′ directionality, including the major cellular nuclease RNase E (64,65). The presence of such a protective hairpin that is disrupted by ChvR binding could thus explain how ChvR represses *chvT* via the upstream binding site. In line with this model, deletion of the 5′ region of the *chvT* transcript (as in *chvT-del5::gfp*) reduces basal expression of the reporter in the absence of ChvR by approximately one third (Supplementary Figure S8). While the exact molecular mechanism of ChvR regulation still awaits experimental validation, the use of complementary approaches targeting distinct aspects of RNA biology could serve as a paradigm for effective gene regulation.

### Regulation by ChvR is independent of Hfq

Hfq contains three principal sites that interact with RNA: the proximal and distal surfaces of the hexameric ring structure, and the rim (66). By binding to two different RNAs at once, Hfq acts as a matchmaker to bring together cognate RNA interaction partners (67,68). Thereby, Hfq contributes to both the specificity of the pairing, as well as to the efficiency of forming base-pairing interactions. In contrast, ChvR sRNA post-transcriptionally regulates expression of its cognate target, *chvT* mRNA, independently of Hfq (Figure 6A and B). Given that *C. crescentus* expresses functional Hfq, is there an advantage for not engaging Hfq in ChvR-mediated regulation? One reason could be an increase in robustness of the regulation. Even though the exact *in vivo* concentration of Hfq is uncertain (in *E. coli*, estimates range from 400–10 000 hexamers per cell (69–71)), the total number of all binding-competent RNAs are clearly in molar excess over the protein. To overcome this limitation, RNAs are thought to rapidly cycle on and off of Hfq to maximize the time associated with the RNA binding protein (72). Profiling of Hfq-associated RNAs has shown that individual, highly expressed RNAs are able to influence the pool of bound species (24,73). As a consequence, competition between sRNAs for Hfq can result in displacement of sRNAs from the chaperone, and reduction of their regulatory potential (74,75). In contrast, the functionality of Hfq-independent sRNAs, like ChvR, is not affected by fluctuation in the transcriptomic output of the cell. Thus, regulation via this class of sRNAs controls gene expression robustly independent of other cellular activities.

While Hfq-independent sRNA regulation may have the benefit of being insulated from the expression levels of other sRNAs, there may also be a cost associated with this type of regulation. Specifically, since Hfq stabilizes RNA–RNA interactions, Hfq-independent regulation may require far more stable associations, potentially explaining why the ChvR:*chvT* interaction involves two distinct sites that include an unusually-long 12 bp continuous homology region. Co-evolving such stable interaction sites may be difficult, which also potentially explains why ChvR has only a single target. Considerably less is known about Hfq-independent sRNAs than their Hfq-dependent counterparts, but our findings with ChvR suggest that sRNA-mediated gene regulation may have initiated through strong base-pairing associations that were later relaxed upon stabilization by Hfq. In extant cases, the trade-off between maintaining strong interactions and insulation from other sRNA levels could dictate which sRNAs use which mechanisms for regulation. In this context, ChvR could represent a useful model for studying sRNA-controlled gene regulation in the absence of Hfq.

## DATA AVAILABILITY

Files of the raw, de-multiplexed reads of *C. crescentus* transcriptomes upon MMC treatment are accessible via the GEO accession GSE104186 (http://www.ncbi.nlm.nih.gov/geo/query/acc.cgi?acc=GSE104186). Microarray data are available from PUMAdb (https://puma.princeton.edu/cgi-bin/publication/viewPublication.pl?pub_no=580).

## Supplementary Material

Supplementary Data
