# Supplementary material for: Post-transcriptional gene regulation by an Hfq-independent small RNA in Caulobacter crescentus
Source: Nucleic Acids Res. 2018 Aug 27;46(20):10969–82. doi: 10.1093/nar/gky765 (PMC6237742; doi:10.1093/nar/gky765)
Supplement: Supplementary Data [file gky765_supplemental_files.pdf]

Supplemental Information

Post-transcriptional gene regulation by an Hfq-independent small RNA in *Caulobacter crescentus*

Kathrin S. Fröhlich, Konrad U. Förstner, and Zemer Gitai

This Supplement contains:

Supplementary Figures S1-S9

Supplementary Tables S1-S4

Supplementary References

Supplementary Figure S1

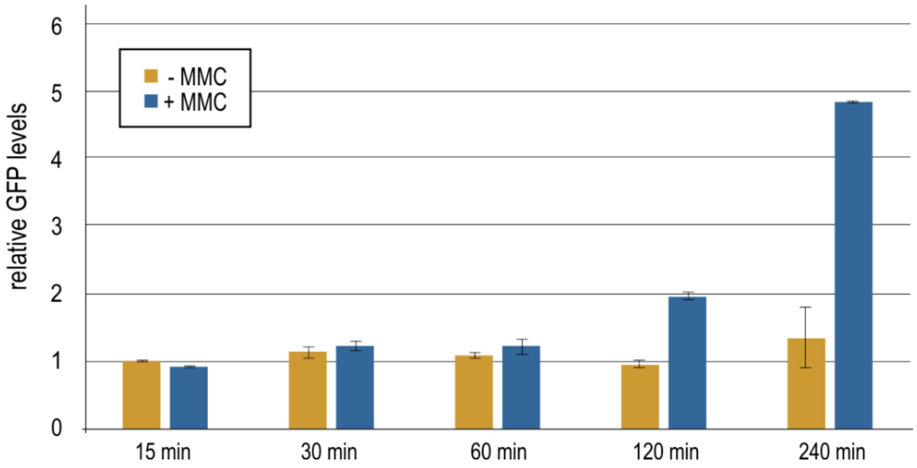

GFP fluorescence of *C. crescentus* carrying the reporter plasmid pP*bapE::gfp* (pKF310-3) at different time-points after addition of the DNA damaging agent MMC to cells grown in PYE (OD_{660} of 0.4 at t=0 min; blue bars). An untreated culture aliquot was sampled in parallel, and served as negative control (yellow bars). Fluorescence levels were normalized to a culture sample collected prior to MMC addition. Error bars indicate the standard deviation calculated of three biological replicates

Supplementary Figure S2

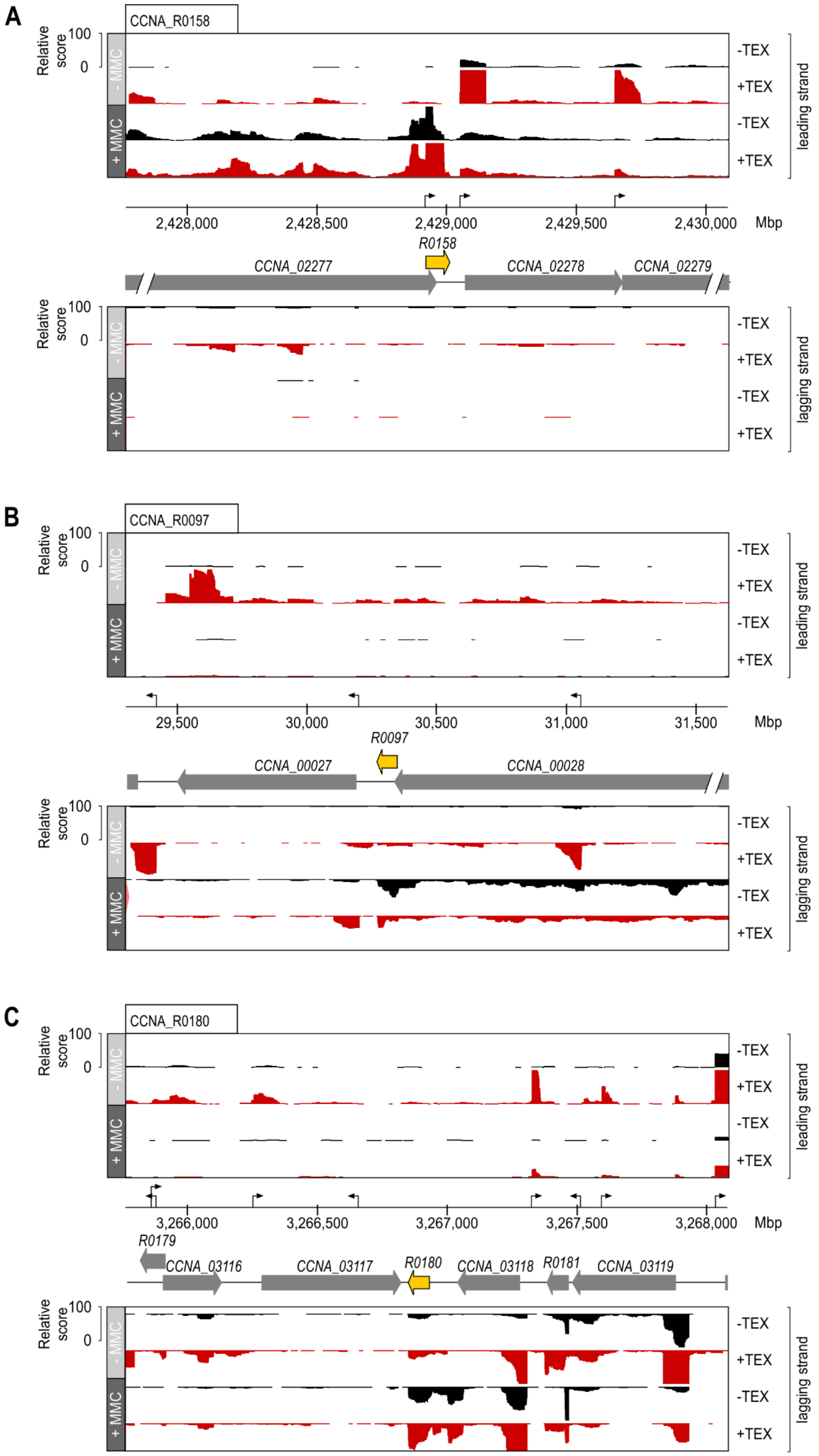

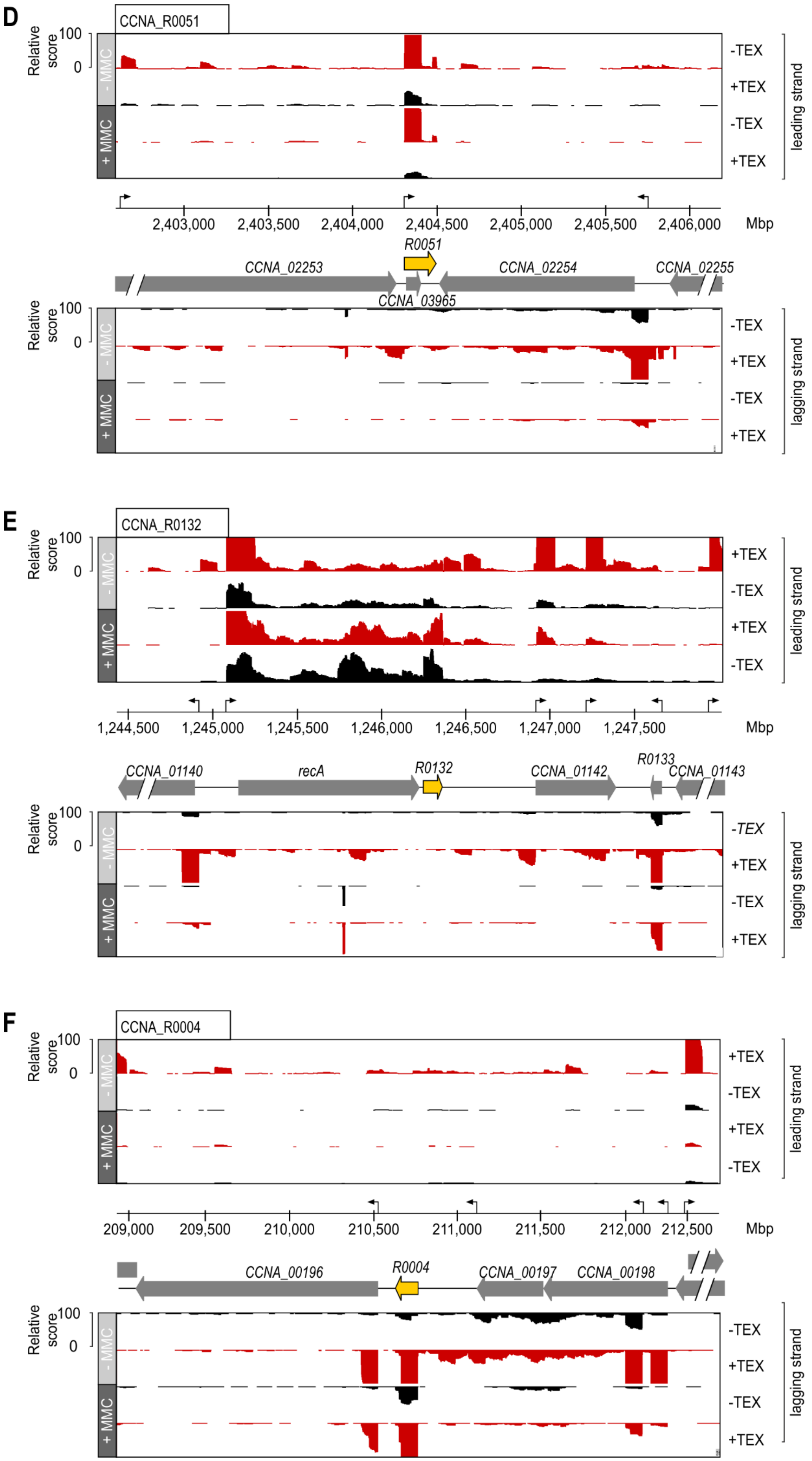
D
CCNA_R0051
Relative score
100
0
- MMC
+ MMC
-TEX
+TEX
-TEX
+TEX
leading strand
2,403,000
2,403,500
2,404,000
2,404,500
2,405,000
2,405,500
2,406,000
Mbp
R0051
CCNA_02253
CCNA_02254
CCNA_02255
CCNA_03965
lagging strand
E
CCNA_R0132
1,244,500
1,245,000
1,245,500
1,246,000
1,246,500
1,247,000
1,247,500
CCNA_01140
recA
R0132
CCNA_01142
R0133
CCNA_01143
F
CCNA_R0004
209,000
209,500
210,000
210,500
211,000
211,500
212,000
212,500
CCNA_00196
R0004
CCNA_00197
CCNA_00198

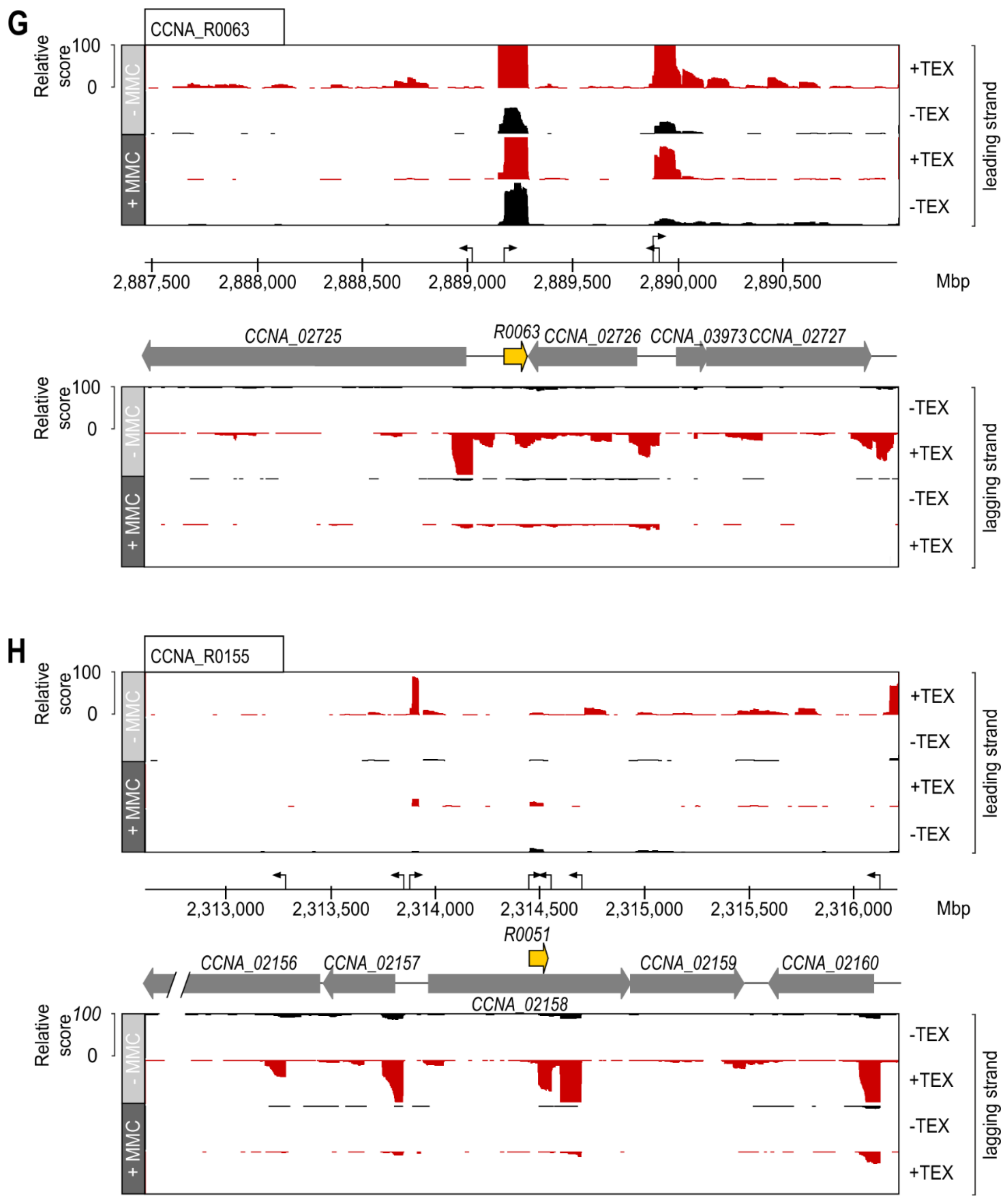

Global transcriptome analysis of the *C. crescentus* response to DNA damage. RNA was collected from *C. crescentus* grown in PYE prior to (OD_{660} of 0.4) or 4h after treatment with the DNA-damaging agent MMC, and analyzed by dRNA-seq. cDNA reads of –TEX (black) and +TEX (red) libraries were mapped to the *C. crescentus* NA1000 genome, and genomic loci encoding candidate sRNAs CCNA_R0158 (A), CCNA_R0097 (B), CCNA_R0180 (C), CCNA_R0051 (D), CCNA_R0132 (E), CCNA_R0004 (F), CCNA_R0063 (G), and CCNA_R0155 (H) are shown. All libraries were adjusted to same scale. The genome coordinates are indicated in the centre. sRNA genes are indicated by yellow arrows, flanking genes are indicated by gray arrows. Transcriptional start sites are marked by black arrows.

Supplementary Figure S3

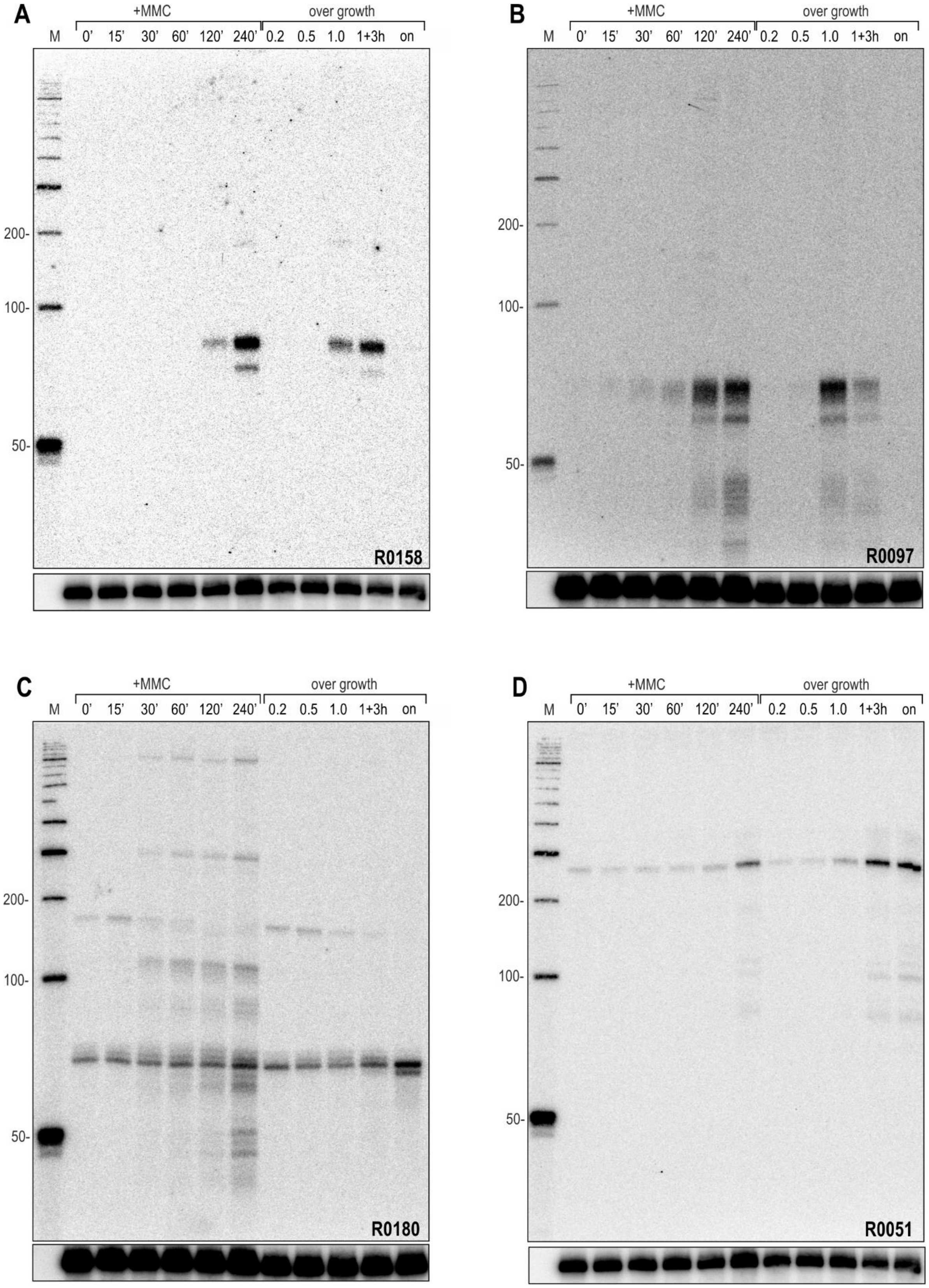

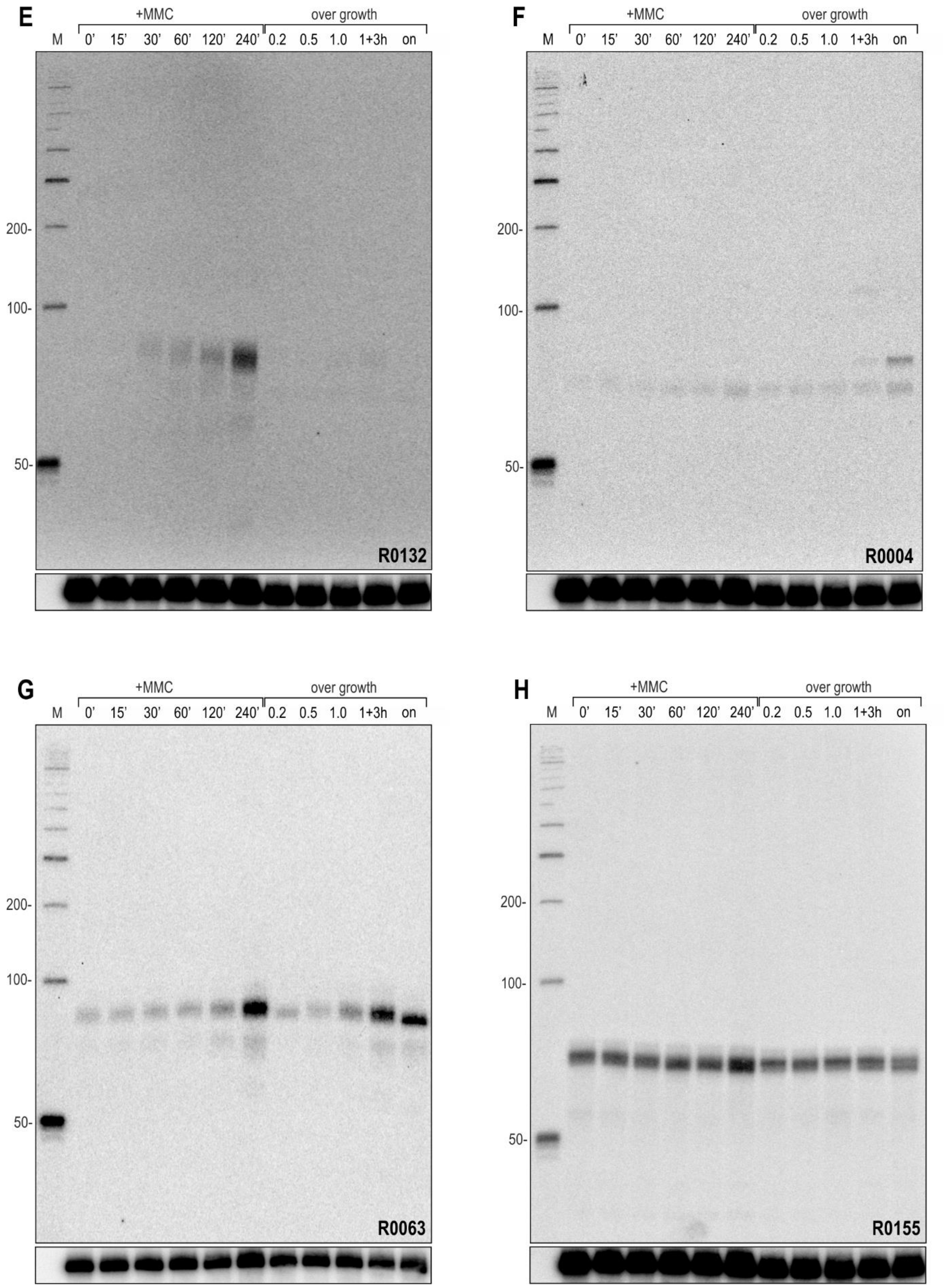

Expression of candidate sRNAs in response to DNA damage. RNA was extracted from wild-type *C. crescentus* prior to and at indicated time-points after addition of MMC to cultures grown in PYE (OD_{660} of 0.4), or from cells harvested at different stages of growth in PYE (OD_{660} of 0.2/0.5/1.0/3h after cells had reached OD_{660} of 1.0/24h). The expression of indicated candidate sRNAs was analyzed on Northern blots hybridized with gene-specific oligo probes. 5S ribosomal RNA served as a loading control.

Supplementary Figure S4

CCNA_00157

...TACAAGCTGGGCTGAaatctcgaagaacagcggattcttaattcgcatccatgaa

agggccccgcagcgatttcgccatggtctcgccacagagtggcggcgagcttggccgc

chvR

GGCGGGGCCTACATGTCGCGCAAGTCTCCGGAGGGCCATTCCGAGGCGGGGCCCGCTC

CAGCGTCCTCATTGGAGCGGGCGTCTcggcccgcctagccccgggggccgacaccgct

recF

taagaacgcccgc**ATGGCGTCGGCCGCACTGCTTTCCCTGACCCTGGCGGACTTT...**

Location of *chvR* (originally annotated as CCNA_R0100) in the genome of *C. crescentus* NA1000. Transcriptional start sites are indicated by arrows, and have been confirmed by transcriptomic data (this study and (1)). The flanking genes CCNA_00157 and *recF* are indicated in gray and yellow, respectively, and translational start and stop codons are underlined.

Supplementary Figure S5

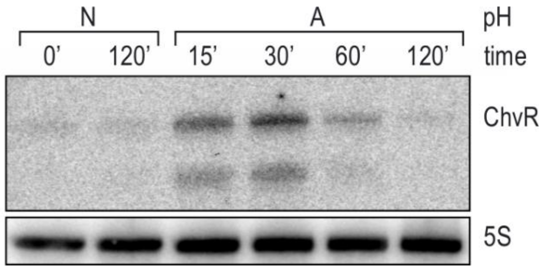

Expression of ChvR in acidified growth medium. *C. crescentus* was grown in PYE to mid-exponential phase when cultures were split, and growth was continued in PYE at either neutral (N; pH=7) or acidic pH (A; pH=5.5). RNA samples were collected at indicated time-points, and ChvR levels were determined by Northern blot analysis.

Supplementary Figure S6

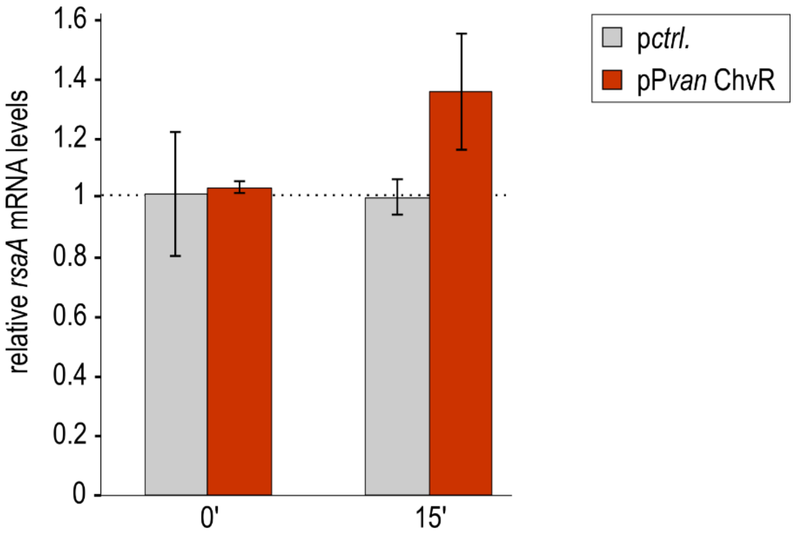

Expression of *rsaA* mRNA in the presence and absence of ChvR sRNA. *C. crescentus* carrying either control plasmid p*ctrl.* (pBVMCS-6; gray bars), or pPvan ChvR (pKF382-1; red bars) were grown in M2G medium to mid-exponential phase (OD_{660} of 0.6). RNA samples were collected prior to and 15 minutes after induction of ChvR expression by addition of vanillate. Abundance of *rsaA* mRNA was determined by qRT-PCR; error bars represent the standard deviation of two biological replicates.

Supplementary Figure S7

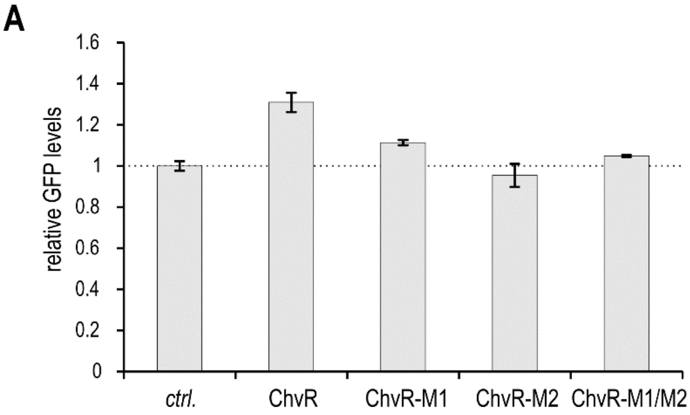

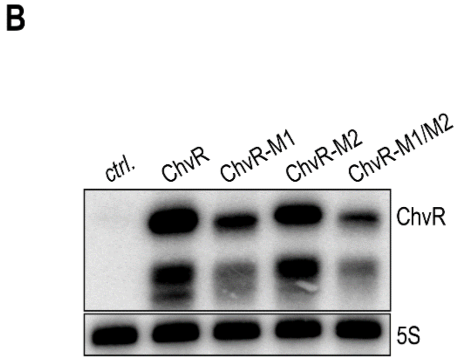

(A) Analysis of GFP production of *C. crescentus* Δ*vanAB* cells expressing fusion *rsaA::gfp* (pKF385-2) in combination with either a control vector (pBVMCS-6), or plasmids expressing ChvR (pKF382-1); ChvR-M1 (pKF395-1); ChvR-M2 (pKF414-1) or ChvR-M1/M2 (pKF418-1) from the inducible P_{van} promoter. Cells were grown overnight in PYE in the presence of vanillate. Error bars represent the standard deviation of three biological replicates. (B) Expression of ChvR variants was determined by Northern blot analysis of RNA samples collected from cells grown as described in (A). An oligo probe recognizing all sRNA versions (KFO-0512) was used for hybridization. 5S ribosomal RNA served as a loading control.

Supplementary Figure S8

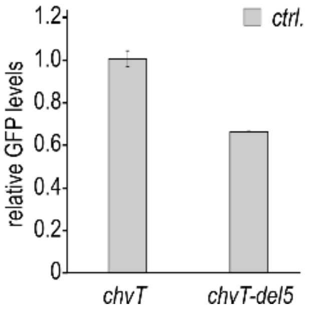

Analysis of GFP production of *C. crescentus* Δ*vanAB* cells expressing fusion *chvT::gfp* (pKF386-1) or *chvT-del5::gfp* (pKF402-1) in combination with a control vector (pBVMCS-6). Cells were grown overnight in PYE in the presence of vanillate. Expression levels of the wild-type fusion were set to 1. Error bars represent the standard deviation of three biological replicates.

Supplementary Figure S9

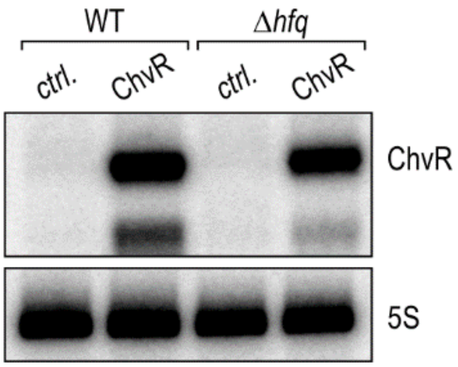

Expression of ChvR was determined by Northern blot analysis of RNA samples collected from *C. crescentus* Δ*vanAB* (WT) or *C. crescentus* Δ*vanAB* Δ*hfq* (Δ*hfq*) cells expressing fusion *chvT::gfp* (pKF386) in combination with either a control vector (pBVMCS-6), or plasmids expressing ChvR (pKF382-1). 5S ribosomal RNA served as a loading control.

Supplementary Table S1 - Genes upregulated at least 3-fold upon treatment with MMC

Locus ID NA1000	Locus ID CB15	annotation	SOS box	log2 FC	Adj p-value
CCNA_02277	CC_2194	TonB-dependent outer membrane channel	no	6,22	2,80E-19
CCNA_R0158	-	small non-coding RNA	no	6,08	4,03E-16
CCNA_02275	-	ABC transporter, periplasmic component	no	5,62	4,79E-15
CCNA_03940	-	hypothetical protein	no	5,58	3,96E-14
CCNA_03904	-	hypothetical protein	no	5,52	1,79E-14
CCNA_02274	CC_2192	EF-Hand domain protein	no	5,32	2,63E-12
CCNA_01476	CC_1410	CRP-family transcription regulator FtrB	no	5,03	3,54E-14
CCNA_00028	CC_0028	TonB-dependent receptor	no	4,83	3,80E-13
CCNA_00619	CC_0583	succinylarginine dihydrolase	no	4,79	2,44E-12
CCNA_00754	CC_0717	Nodulin-21 / CCC1-related protein	no	4,78	1,40E-11
CCNA_00617	CC_0581	arginine N-succinyltransferase, beta chain	no	4,77	3,30E-13
CCNA_00618	CC_0582	succinylglutamic semialdehyde dehydrogenase	no	4,75	9,63E-14
CCNA_03826	CC_3711	conserved hypothetical protein	no	4,59	2,44E-12
CCNA_03065	CC_2970	cytochrome c family protein	no	4,56	1,15E-07
CCNA_01466	CC_1400	hypothetical protein	no	4,53	8,60E-07
CCNA_02452	CC_2367	hypothetical protein	no	4,52	1,09E-10
CCNA_01467	CC_1401	cytochrome cbb3 oxidase subunit I ccoN	no	4,50	1,12E-09
CCNA_02604	CC_2519	host cell attachment protein	no	4,49	3,61E-07
CCNA_00154	CC_0155	conserved hypothetical membrane protein	no	4,46	5,97E-07
CCNA_00620	CC_0584	acetylornithine aminotransferase/succinyldiaminopimelate aminotransferase	no	4,42	2,44E-12
CCNA_R0097	-	small non-coding RNA	no	4,27	8,92E-07
CCNA_02610	CC_2526	glyoxalase family protein	no	4,22	1,15E-07
CCNA_00800	CC_0762	cytochrome bd-type quinol oxidase, subunit 1 cydA	no	4,20	8,70E-10
CCNA_01477	CC_1411	oxygen-independent coproporphyrinogen-III oxidase hemN	no	4,18	3,61E-10
CCNA_00789	CC_0752	hypoxia transcriptional regulator FixK	no	4,18	7,68E-07
CCNA_03933	-	hypothetical protein	no	4,15	1,46E-07
CCNA_03932	-	hypothetical protein	no	4,10	1,84E-07
CCNA_03825	CC_3710	hypothetical protein	no	4,09	3,59E-09
CCNA_03155	CC_3059	PepSY-associated transmembrane protein	no	4,03	2,60E-08
CCNA_00279	CC_0277	NAD(P)H-quinone reductase	no	4,01	9,42E-09
CCNA_00434	CC_0425	conserved membrane-spanning protein	no	3,86	1,40E-05
CCNA_01980	CC_1903	PEP phosphonomutase related enzyme	no	3,81	1,17E-07
CCNA_03400	CC_3291	conserved hypothetical protein	no	3,78	4,32E-07
CCNA_02418	CC_2333	uracil DNA glycosylase superfamily protein	yes	3,74	8,56E-07
CCNA_02396	CC_2311	GST / metaxin-like protein	no	3,73	1,46E-07
CCNA_03318	CC_3212	DNA polymerase Y-family protein	no	3,70	5,95E-05
CCNA_00027	CC_0027	2OG-Fe(II) oxygenase	no	3,67	2,99E-07
CCNA_01391	CC_1330	radical SAM superfamily protein	yes	3,65	2,84E-06
CCNA_00799	CC_0761	ABC transporter, ATP-binding protein cydD	no	3,64	9,47E-06
CCNA_03022	CC_2927	PiuB-family transmembrane transporter	no	3,55	1,87E-06
CCNA_00748	CC_0711	ferrous iron transport protein A	no	3,52	1,28E-06

CCNA_03159	CC_3063	sulfite reductase (NADPH) flavoprotein alpha-component	no	3,52	3,82E-05
CCNA_03157	CC_3061	conserved hypothetical protein	no	3,47	3,83E-04
CCNA_00663	CC_0627	bacterial apoptosis endonuclease BapE	yes	3,47	2,63E-05
CCNA_01520	CC_1453	conserved hypothetical protein	no	3,40	1,45E-05
CCNA_00749	CC_0712	ferrous iron transport protein B	no	3,40	8,56E-07
CCNA_00433	-	type 1 capsular polysaccharide biosynthesis protein J	no	3,40	8,66E-05
CCNA_03118	CC_3023	hypothetical protein	no	3,40	2,38E-04
CCNA_00276	CC_0274	conserved hypothetical protein	no	3,37	1,74E-05
CCNA_03840	CC_3724	succinyl-CoA:coenzyme A transferase	no	3,37	3,54E-05
CCNA_02876	-	very short patch repair (Vsr) endonuclease	no	3,36	1,22E-05
CCNA_01767	CC_1696	cytochrome C family protein	no	3,34	2,80E-05
CCNA_01475	CC_1409	OmpW family outer membrane protein	no	3,33	1,39E-04
CCNA_02615	CC_2532	homogentisate 1,2-dioxygenase	no	3,28	1,41E-06
CCNA_03158	CC_3062	iron-sulfur cluster assembly/repair protein ApbE	no	3,28	4,12E-04
CCNA_03466	CC_3356	ORF6N domain protein	yes	3,26	1,69E-04
CCNA_03317	CC_3211	DNA polymerase III alpha subunit	no	3,23	4,00E-05
CCNA_00798	CC_0760	ABC transporter, ATP-binding protein cydC	no	3,21	1,11E-03
CCNA_02603	CC_2518	conserved hypothetical protein	no	3,19	1,29E-04
CCNA_02616	CC_2533	4-hydroxyphenylpyruvate dioxygenase	no	3,15	9,21E-06
CCNA_02355	CC_2272	endonuclease III	yes	3,15	7,43E-04
CCNA_01519	CC_1452	universal stress protein family	no	3,12	1,53E-05
CCNA_02004	CC_1927	SOS-induced inhibitor of cell division SidA	yes	3,09	8,46E-06
CCNA_02974	CC_2880	toxin protein relE-3	yes	3,06	4,11E-05
CCNA_03537	CC_3424	glyoxalase family protein	yes	3,06	6,38E-06
CCNA_01468	CC_1402	cytochrome cbb3 oxidase, cytochrome c subunit ccoO	no	3,02	2,29E-03
CCNA_01600	CC_1531	Methane oxygenase, PmoA family	yes	3,00	1,33E-05
CCNA_01470	CC_1404	cytochrome cbb3 oxidase, diheme subunit, membrane-bound ccoP	no	2,96	7,92E-04
CCNA_03981	-	hypothetical protein	no	2,94	1,41E-03
CCNA_03372	CC_3263	bacterioferritin-associated ferredoxin	no	2,94	1,25E-03
CCNA_03931	-	hypothetical protein	no	2,89	5,79E-03
CCNA_03319	CC_3213	conserved hypothetical protein	yes	2,88	2,25E-03
CCNA_00801	CC_0763	cytochrome bd-type quinol oxidase, subunit 2 cydB	no	2,85	1,38E-04
CCNA_02433	CC_2348	hypothetical protein	no	2,84	2,17E-03
CCNA_01582	CC_1514	Glutathione-dependent formaldehyde-activating enzyme	no	2,80	2,52E-03
CCNA_00593	CC_0558	conserved hypothetical protein	no	2,77	1,69E-04
CCNA_02614	CC_2531	fumarylacetoacetase superfamily protein	no	2,75	2,60E-04
CCNA_03207	CC_3109	conserved hypothetical protein	no	2,73	4,41E-03
CCNA_02727	CC_2644	phosphate starvation-inducible protein PhoH	no	2,73	2,09E-03
CCNA_00719	CC_0683	type I secretion adaptor protein hlyD	no	2,72	2,19E-03
CCNA_02417	CC_2332	biotin synthase related domain containing protein	yes	2,72	2,89E-04
CCNA_03156	CC_3060	putative periplasmic protein	no	2,70	1,38E-02
CCNA_03629	CC_3514	DNA replication inhibitor toxin SocB	no	2,64	6,34E-05
CCNA_00594	CC_0559	conserved hypothetical protein	no	2,64	2,23E-03
CCNA_02973	CC_2879	antitoxin protein relB-3	yes	2,63	6,91E-04
CCNA_02200	CC_2115	cytochrome c-family protein	no	2,62	8,81E-04
CCNA_02375	CC_2292	hypothetical protein	no	2,55	4,84E-03

CCNA_03023	CC_2928	TonB-dependent receptor	no	2,54	4,09E-03
CCNA_03955	-	hypothetical protein	no	2,53	1,65E-03
CCNA_00426	CC_0421	very-short-patch-repair endonuclease	no	2,52	4,55E-03
CCNA_01149	CC_1093	TonB-dependent outer membrane receptor	no	2,48	2,19E-03
CCNA_01471	CC_1405	polyferredoxin protein fixG	no	2,46	2,02E-02
CCNA_01141	CC_1087	RecA protein	yes	2,46	2,58E-04
CCNA_01766	CC_1695	CsgG-related curli protein	no	2,44	4,66E-04
CCNA_02613	CC_2530	Rieske (2Fe-2S) domain protein	no	2,44	4,37E-03
CCNA_00386	CC_0382	DNA-3-methyladenine glycosylase	yes	2,43	1,65E-03
CCNA_00766	CC_0729	cobalamin adenosyltransferase family protein	no	2,41	1,31E-03
CCNA_01107	-	toxin protein parE-2	no	2,39	6,81E-03
CCNA_00854	CC_0811	metallo-beta-lactamase protein	no	2,35	5,26E-02
CCNA_03245	CC_3143	beta alanine-pyruvate transaminase	no	2,34	1,15E-02
CCNA_01106	CC_1054	antitoxin protein parD-2	yes	2,33	5,26E-02
CCNA_03660	-	Usg protein	no	2,31	5,43E-03
CCNA_02617	CC_2534	histidinol-phosphate aminotransferase	no	2,27	1,84E-02
CCNA_03141	CC_3046	phosphoserine phosphatase	no	2,27	1,75E-02
CCNA_03930	-	hypothetical protein	no	2,26	5,43E-03
CCNA_03633	CC_3518	GIY-YIG domain protein	yes	2,24	1,75E-02
CCNA_00853	CC_0810	3-isopropylmalate dehydrogenase	no	2,22	2,72E-02
CCNA_00745	CC_0708	conserved hypothetical protein	no	2,21	4,15E-02
CCNA_R0180	-	small non-coding RNA	no	2,20	1,02E-01
CCNA_00767	CC_0730	conserved hypothetical protein	no	2,20	1,12E-02
CCNA_01599	CC_1530	conserved hypothetical protein	no	2,20	5,66E-02
CCNA_R0100	-	small non-coding RNA	no	2,17	1,17E-01
CCNA_00720	CC_0684	type I protein secretion ATP-binding protein/type I protein secretion transmembrane subunit/type I secretion processing peptidase	no	2,13	3,04E-02
CCNA_01708	CC_1636	conserved hypothetical protein	no	2,10	5,92E-02
CCNA_R0051	-	small non-coding RNA	no	2,10	1,37E-01
CCNA_03630	CC_3515	antitoxin protein SocA	yes	2,09	4,37E-03
CCNA_02283	CC_2200	HNH endonuclease family protein	no	2,07	1,25E-02
CCNA_03965	-	hypothetical protein	no	2,06	1,51E-01
CCNA_03343	CC_3235	FtsW related protein	no	2,06	1,42E-02
CCNA_03886	-	trypsin-like peptidase domain family protein	no	2,05	7,31E-02
CCNA_03242	CC_3140	succinic semialdehyde dehydrogenase	no	2,03	2,44E-02
CCNA_02779	CC_2696	LysM family peptidoglycan binding protein	no	2,03	1,06E-02
CCNA_03982	-	hypothetical protein	no	2,03	2,02E-02
CCNA_R0132	-	small non-coding RNA	no	2,03	1,57E-01
CCNA_02831	CC_2745	conserved hypothetical protein	no	2,01	9,50E-03
CCNA_00106	CC_0107	2-polyprenyl-6-methoxyphenol hydroxylase	no	2,01	2,57E-02
CCNA_03232	CC_3131	toxin protein relE-4	no	2,01	1,77E-02
CCNA_03231	-	anti-toxin protein relB-4	no	2,00	1,13E-02
CCNA_00930	CC_0886	riboflavin synthase alpha chain	no	2,00	3,89E-02
CCNA_00802	-	cyd operon protein YbgT	no	1,98	4,60E-02
CCNA_00138	CC_0139	TonB-dependent receptor	no	1,98	2,23E-02
CCNA_01981	CC_1904	DNA translocation competence protein ComA	no	1,97	4,43E-02

CCNA_01979	CC_1902	LexA repressor	yes	1,97	5,82E-02
CCNA_01016	CC_0965	copper resistance protein B	no	1,97	5,64E-02
CCNA_03929	-	hypothetical protein	no	1,96	5,54E-02
CCNA_00592	CC_0557	ferritin superfamily protein	no	1,94	3,03E-02
CCNA_R0004	-	cell cycle regulated sRNA	no	1,93	1,79E-01
CCNA_03962	-	hypothetical protein	no	1,92	1,72E-01
CCNA_02312	CC_2229	TraB superfamily protein	no	1,91	1,42E-02
CCNA_03313	CC_3207	hypothetical protein	no	1,91	1,11E-01
CCNA_03342	CC_3234	short-chain acyl-CoA hydrolase	no	1,90	4,36E-02
CCNA_00967	CC_0919	TetR-family transcriptional regulator	no	1,89	9,31E-02
CCNA_00932	CC_0888	6,7-dimethyl-8-ribityllumazine synthase	no	1,88	1,15E-01
CCNA_01848	CC_1770	quinol cytochrome oxidase polypeptide IV	no	1,87	5,92E-02
CCNA_01847	CC_1769	cytochrome c oxidase assembly protein Surf1	no	1,87	3,68E-02
CCNA_00931	CC_0887	GTP cyclohydrolase II/3,4-dihydroxy-2-butanone-4-phosphate synthase	no	1,86	1,84E-02
CCNA_02554	CC_2469	conserved hypothetical protein	no	1,84	7,31E-02
CCNA_03986	-	hypothetical protein	no	1,84	6,88E-02
CCNA_R0063	-	minimal medium expressed sRNA	no	1,82	1,97E-01
CCNA_03580	CC_3467	conserved hypothetical protein	yes	1,81	1,04E-01
CCNA_00622	-	hypothetical protein	no	1,81	6,30E-02
CCNA_01409	CC_1347	delta-aminolevulinic acid dehydratase	no	1,81	2,54E-02
CCNA_03695	CC_3580	aldehyde dehydrogenase	no	1,80	6,30E-02
CCNA_03346	CC_3238	crossover junction endodeoxyribonuclease ruvC	yes	1,80	7,31E-02
CCNA_01491	CC_1424	cystathionine beta-lyase	no	1,80	5,09E-02
CCNA_R0046	-	tRNA	no	1,79	2,34E-01
CCNA_03928	-	hypothetical protein	no	1,78	8,78E-02
CCNA_03243	CC_3141	NADP+-dependent gamma-glutamyl-gamma-aminobutyraldehyde dehydrogenase	no	1,78	6,55E-02
CCNA_R0054	-	tRNA	no	1,78	2,30E-01
CCNA_01492	CC_1425	3-mercaptopyruvate sulfurtransferase	no	1,76	7,21E-02
CCNA_02812	CC_2727	glutaredoxin	no	1,76	1,64E-01
CCNA_00613	CC_0577	cyanophycinase	no	1,75	9,81E-02
CCNA_00029	CC_0029	lysine exporter protein	no	1,75	8,60E-02
CCNA_01849	CC_1771	quinol cytochrome oxidase polypeptide III	no	1,75	1,28E-01
CCNA_01610	CC_1541	2-isopropylmalate synthase	no	1,74	5,92E-02
CCNA_02397	CC_2312	DeoR-family transcriptional regulator	no	1,74	1,51E-01
CCNA_R0027	-	tRNA	no	1,74	2,41E-01
CCNA_01457	CC_1391	hypothetical protein	no	1,74	1,67E-01
CCNA_03815	CC_3701	GumN superfamily protein	no	1,73	6,03E-02

Supplementary Table S2 - Oligonucleotides

Oligo ID	Sequence 5' to 3'	description
KFO-0026	GTTTTGGATCCTCTAGAAGTAAAGGTGAAGAACTGTTC	construction of plasmid pKF308-1
KFO-0028	GTTTTTGAGCTCTTATTTGTAGAGTTCATCCATG	construction of plasmid pKF308-1
KFO-0029	GTTTTTCTCGAGGGGCTGTAAGGACCACTT	construction of plasmid pKF310-3
KFO-0035	GTTTTTTCTAGACGCAGCGTTGGCGGACA	construction of plasmid pKF310-3
KFO-0056	AAGCTTGATATCGAATTCCTGCA	amplification of pBVMCS-6/pBXMCS-6
KFO-0059	CGAATTCGTGGATCCAGATATC	amplification of pNPTS138
KFO-0060	CTTCGGCCGTGACGCGTCT	amplification of pNPTS138
KFO-0065	GATATCTGGATCCACGAATTCGAACAGCGCTTCGCCCTCG	construction of plasmid pKF323-3
KFO-0066	ACGCGGGGCCTTCGTCGTGGTTTCGGTACCCATCGTC	construction of plasmid pKF323-3
KFO-0067	GACGATGGGTACCGAAACCACGACGAAGGCCCCGCGT	construction of plasmid pKF323-3
KFO-0068	AGACGCGTCACGGCCGAAGTGATGAGCTTGCCGCTGTTG	construction of plasmid pKF323-3
KFO-0069	GATATCTGGATCCACGAATTCGCGACGACATGGGCCTGGT	construction of plasmid pKF416-15
KFO-0072	AGACGCGTCACGGCCGAAGCGCTCATAGTCGAGCCTGG	construction of plasmid pKF416-15
KFO-0113	CAGGGGGACTTAACGACCGAGTTC	oligo probe for 5S ribosomal RNA
KFO-0144	GGATCCAATCTTGATCGTAAT	amplification of pBVMCS-6
KFO-0221	ATGGCCCTCCGGAGACTTG	oligo probe for ChvR RNA
KFO-0222	ATGGGCTCCGCCCGAAGAG	oligo probe for CCNA_R0004 RNA
KFO-0227	GTTTTCTGCAGTCTCGAAGAACAGCGGATTC	construction of plasmid pKF370-1
KFO-0230	GTTTTTCTAGAGGTCAGGGAAAGCAGTG	construction of plasmid pKF370-1; pKF382-1
KFO-0231	P-GGCGGGGCCTACATGTCG	construction of plasmid pKF382-1
KFO-0252	GATATCTGGATCCACGAATTCGATGGCCAAGCTGAACTATGTG	construction of plasmid pKF379-1
KFO-0253	GGCCGAGACGCCCGCTCCAACCACTCTGTGGCGAGACCAT	construction of plasmid pKF379-1
KFO-0254	ATGGTCTCGCCACAGAGTGGTTGGAGCGGGCGTCTCGGCC	construction of plasmid pKF379-1
KFO-0255	AGACGCGTCACGGCCGAAGGTCAGCCAGATCGGCCGCA	construction of plasmid pKF379-1
KFO-0277	GTTCTTCGAGATTTCAGGCTAGCTGCAGCCCGGGGG	construction of plasmid pKF383-7
KFO-0278	ATAAAGCTGACCCTATGCATATTAATTAAGGCGCCTGC	construction of plasmid pKF384-1
KFO-0282	CCCGGGCTGCAGCTAGCCTGAAATCTCGAAGAACAGCGGA	construction of plasmid pKF383-7
KFO-0283	ATGCGTTTCCTCGCATCGTGGAGCTCAGGCCCCGCCGCGGCCAA	construction of plasmid pKF383-7
KFO-0286	GCGCTTCAGACGCTCGAGTTATTTTTGACACCAGACCAAC	construction of plasmid pKF383-7
KFO-0287	GTTGGTCTGGTGTCAAAAATAACTCGAGCGTCTGAAGCGC	construction of plasmid pKF383-7
KFO-0289	GATGCGAGGAAACGCATATGCCCGTCGTTTTACAACGTCG	construction of plasmid pKF383-7
KFO-0313	GAGTACGAGAAGAACGACGAAG	amplification of *chvT* for qRT PCR
KFO-0314	CTGATCAGGATGTTGTCCAGAA	amplification of *chvT* for qRT PCR
KFO-0315	TCCAGACCTACCAGTTCTTCAC	amplification of *rsaA* for qRT PCR
KFO-0316	CCTGAGCGAACTTCGAGTAGTA	amplification of *rsaA* for qRT PCR
KFO-0317	GCGGACTTTCGGTCCTAC	amplification of *recF* for qRT PCR
KFO-0318	TGATGGCTTCGAGGAGATTG	amplification of *recF* for qRT PCR
KFO-0319	GAGCAGCTCTATGCGATCAA	amplification of *rpoD* for qRT PCR
KFO-0320	GTTCAGCTCCGATCCGAAATA	amplification of *rpoD* for qRT PCR
KFO-0321	CTTTTTAGATCTATGGGTACCGTGAGCAAGGGCGAGGAG	construction of plasmid pKF384-1
KFO-0323	GTTTTTAGATCTGAATTCGCTATAGAGCAAACGTCATACG	construction of plasmid pKF384-1
KFO-0326	CTTTTGAATTCGAGGAATGGCCCTCTTTGC	construction of plasmid pKF386-1
KFO-0327	CTTTTGGTACCTCCCAGCATCGTCGTCGC	construction of plasmid pKF386-1; pKF397-4; pKF402-1
KFO-0331	GTTTTTCATATGAGGCATGGTCGACAGCAGC	construction of plasmid pKF384-1
KFO-0336	ACTACAAGGACCACGACATCGACTACAAGGACGACGACGACAAGTAGTAAGCCGAGACCTTCGGGTCAA	construction of plasmid pKF436-1
KFO-0337	TTGTAGTCGATGTCGTGGTCCTTGTAGTCGCCGTCGTGGTCCTTGTAGTCCATCTTGTAGTTGAAGCCGAG	construction of plasmid pKF436-1
KFO-0338	GATATCTGGATCCACGAATTCGATCGACTTCCAGTTCCTGTACC	construction of plasmid pKF436-1
KFO-0339	AGACGCGTCACGGCCGAAGTGTTGAGGACGAGATCGCCG	construction of plasmid pKF436-1
KFO-0345	GATATCTGGATCCACGAATTCGTGTAGAGATCGGCGGGCTG	construction of plasmid pKF389-6
KFO-0346	GCGGTCTTGATACGCCCGTCATCGGGTCGATACGTGTCC	construction of plasmid pKF389-6
KFO-0347	GGACACGTATCGACCCGATGACGGGCGTATCAAGACCGC	construction of plasmid pKF389-6
KFO-0348	AGACGCGTCACGGCCGAAGTTCTCCGGCGAGCACGTAG	construction of plasmid pKF389-6
KFO-0349	CTTAGGCTAGCATGCCCTACCCGCTCCAGG	construction of plasmid pKF390-14
KFO-0350	CTTAGCATATGGCCTTGTATCTCAAACGGGATC	construction of plasmid pKF390-14
KFO-0354	TCCGGAGGCCCATTCCGAGGCGGGGCCC	construction of plasmid pKF395-1
KFO-0355	CTCGGAATGGGCCTCCGGAGACTTGCGC	construction of plasmid pKF395-1
KFO-0372	CTTTTGAATTCGAGGAATGGGCCTCTTTGCTCCCGCGC	construction of plasmid pKF397-4
KFO-0382	CTTTTGAATTCTTTGCTCCCGCGCGCGGC	construction of plasmid pKF402-1
KFO-0417	CCAGCGTCGTCATTGGAGCGGGCGTCTC	construction of plasmid pKF414-1; pKF418-1
KFO-0418	TCCAATGACGACGCTGGAGCGGGCCC	construction of plasmid pKF414-1; pKF418-1
KFO-0426	ACTTTTGACGACAACAAAAACATGGCGTTG	construction of plasmid pKF420; pKF466-1

KFO-0427	TTGTTGTCGTCAAAAGTACGCGCGCCC	construction of plasmid pKF420; pKF466-1
KFO-0476	TGTCAGGGGAGGCTCCGCG	oligo probe for CCNA_R0180 RNA
KFO-0477	GTCACGACTTGCGACCGCG	oligo probe for CCNA_R0051 RNA
KFO-0480	TGTAGTTCCGGCCAGGCTG	oligo probe for CCNA_R0158 RNA
KFO-0481	GGCAGGGTGGCGCTAAGC	oligo probe for CCNA_R0155 RNA
KFO-0482	TGGGCACATGAGCGCGAG	oligo probe for CCNA_R0063 RNA
KFO-0483	CTTTTGAATTCTGCTCCCATGCGCGCCAC	construction of plasmid pKF385-2
KFO-0484	CTTTTGGTACCGGCGTTGGTGTACGCAGTC	construction of plasmid pKF385-2
KFO-0503	AGGCGGCCTGAGATCGCG	oligo probe for CCNA_R0132 RNA
KFO-0504	ACAGGTCGGACGGGGCG	oligo probe for CCNA_R0097 RNA
KFO-0512	GGCCCCGCCTCGGAATG	oligo probe for ChvR RNA (all variants)
KFO-0579	GGGCCAGTTCATTAGGGAGG	oligo probe for CCNA_R0014 RNA

Supplementary Table S3 - Plasmids

trivial name	plasmid ID	description	backbone/marker	reference
pctrl.	pBVMCS-6	empty vector	pBVMCS-6/CmR	(2)
	pBXMCS-6	empty vector	pBXMCS-6/CmR	(2)
	pGFPC-2	empty vector	pGFPC-2 /KanR	(2)
	pNPTS138	empty vector	pNPTS138/KanR	M. R. Alley, unpublished
	pPR9TT	empty vector	pPR9TT/AmpR,CmR	(3)
	pRL27	Transposon delivery vector	pRL27/KanR	(4)
	pVGFP-5	empty vector	pVGFP-5 /TetR	(2)
	pXGFP-5	empty vector	pXGFP-5 /TetR	(2)
	pXG10sf	empty vector	pXG10sf/CmR	(5)
pP*bapE::gfp*	pKF310-3	expression of *bapE::gfp* fusion (up to the 10th codon of *bapE*) under control of the *bapE* promoter	pPR9TT/AmpR,CmR	this study
	pKF323-2	allelic replacement of *vanAB*	pNPTS138/KanR	this study
	pKF370-1	expression of *chvR* under control of the native promoter	pBXMCS-6/CmR	this study
	pKF379-1	allelic replacement of *chvR*	pNPTS138/KanR	this study
pVan-ChvR	pKF382-1	expression of ChvR under control of the *van* promoter	pBVMCS-6/CmR	this study
pP*chvR::lacZ*	pKF383-7	expression of *chvR::lacZ* transcriptional fusion (-109 to +9 of chvR relative to TSS); integration into *xyl* locus	pXGFP-5 /TetR	this study
	pKF384-1	empty vector	pGFPC-2 /KanR	this study
rsaA::gfp	pKF385-2	expression of *rsaA::gfp* translational fusion (up to +45 of *rsaA* relative to the translational start site) under control of the *rsaA* promoter; integration into *rsaA* locus	pGFPC-2 /KanR	this study
chvT::gfp	pKF386-1	expression of *chvT::gfp* translational fusion (-72 to +45 of *chvT* relative to the translational start site) under control of the *rsaA* promoter; integration into *rsaA* locus	pGFPC-2 /KanR	this study
	pKF389-6	allelic replacement of *chvIG-hprK*	pNPTS138/KanR	this study
p*chvIG-hprK*	pKF390-14	expression of *chvIG-hprK* operon under control of the native promoter; integration into *xyl* locus	pXGFP-5 /TetR	this study
ChvR-M1	pKF395-1	expression of ChvR-M1 (SNE G35C relative to TSS) under control of the van promoter	pBVMCS-6/CmR	this study
chvT-M1::gfp	pKF397-4	expression of *chvT-M1::gfp* translational fusion (-72 to +45 of *chvT* relative to the translational start site) under control of the *rsaA* promoter; SNE C-63G; integration into *rsaA* locus	pGFPC-2 /KanR	this study
chvT-del5::gfp	pKF402-1	expression of *chvT-del5::gfp* translational fusion (-58 to +45 of *chvT* relative to the translational start site) under control of the *rsaA* promoter; integration into *rsaA* locus	pGFPC-2 /KanR	this study
ChvR-M2	pKF414-1	expression of ChvR-M2 (SNE C66G relative to TSS) under control of the *van* promoter	pBVMCS-6/CmR	this study
	pKF416-15	chromosomal integration of 3XFLAG at *hfq* N-terminus via allelic replacement	pNPTS138/KanR	this study

ChvR-M1M2	pKF418-1	expression of ChvR-M1M2 (SNE G35C and C66G relative to TSS) under control of the *van* promoter	pBVMCS-6/CmR	this study
chvT-M2::gfp	pKF420-1	expression of *chvT-M2::gfp* translational fusion (-72 to +45 of *chvT* relative to the translational start site) under control of the *rsaA* promoter; SNE G-13C; integration into *rsaA* locus	pGFPC-2 /KanR	this study
	pKF436-1	chromosomal integration of 3XFLAG at *chvT* C-terminus via allelic replacement	pNPTS138/KanR	this study
chvT-M1M2::gfp	pKF466-1	expression of *chvT-M1M2::gfp* translational fusion (-72 to +45 of *chvT* relative to the translational start site) under control of the *rsaA* promoter; SNE C-63G and G-13C; integration into *rsaA* locus	pGFPC-2 /KanR	this study

Supplementary Table S4 - Strains

Strain	Stock name	Bacterium	Genotype/relevant markers	Source/reference
wild-type	KFS-0006	*C. crescentus* NA1000		laboratory stock
∆vanAB	KFS-0058	*C. crescentus* NA1000	*∆vanAB*	this study
	KFS-0143	*C. crescentus* NA1000	*∆lacA*	JOE2321; (6)
∆chvR	KFS-0150	*C. crescentus* NA1000	*∆chvR*	this study
∆lacA	KFS-0172	*C. crescentus* NA1000	*∆lacA xylX::pKF383*	this study
∆lacA chvI::Tn5	KFS-0174	*C. crescentus* NA1000	*∆lacA xylX::pKF383 chvI::Tn5(tetR)*	this study
	KFS-0179	*C. crescentus* NA1000	*∆vanAB PrsaA::pKF386-1*	this study
∆chvIG-hprK	KFS-0185	*C. crescentus* NA1000	*∆chvIG-hprK*	this study
∆chvIG-hprK (complemented)	KFS-0189	*C. crescentus* NA1000	*∆chvIG-hprK xylX::pKF390-14*	this study
	KFS-0297	*C. crescentus* NA1000	*3XFLAG::hfq*	UJ10248; Jenal laboratory, Basel University
	KFS-0316	*C. crescentus* NA1000	*∆vanAB PrsaA::pKF402-1*	this study
	KFS-0334	*C. crescentus* NA1000	*∆vanAB PrsaA::pKF420-1*	this study
3XFLAG::hfq	KFS-0344	*C. crescentus* NA1000	*3XFLAG::hfq*	this study
chvT::3XFLAG	KFS-0392	*C. crescentus* NA1000	*chvT::3XFLAG*	this study
chvT::3XFLAG ∆chvR	KFS-0394	*C. crescentus* NA1000	*chvT::3XFLAG ∆chvR*	this study
	KFS-0513	*C. crescentus* NA1000	*∆vanAB PrsaA::pKF385-2*	this study
	KFS-0550	*C. crescentus* NA1000	*∆vanAB PrsaA::pKF466-1*	this study
	KFS-0570	*C. crescentus* NA1000	*∆hfq::tet*	CJWS 5477; (7)
∆hfq	KFS-0796	*C. crescentus* NA1000	*∆hfq::tet*	this study
∆vanAB ∆hfq	KFS-0797	*C. crescentus* NA1000	*∆hfq ∆vanAB*	this study
	KFS-0798	*C. crescentus* NA1000	*∆hfq ∆vanAB PrsaA::pKF386-1*	this study
chvT::3XFLAG ∆hfq	KFS-0911	*C. crescentus* NA1000	*chvT::3XFLAG ∆hfq::tet*	this study
chvT::3XFLAG ∆chvR ∆hfq	KFS-0912	*C. crescentus* NA1000	*chvT::3XFLAG ∆chvR ∆hfq::tet*	this study
	KFS-0013	*E. coli* S17 -1*λpir*		laboratory stock
	KFS-0088	*E. coli* TOP10		Invitrogen

Supplementary References

1. Schrader, J.M., Zhou, B., Li, G.W., Lasker, K., Childers, W.S., Williams, B., Long, T., Crosson, S., McAdams, H.H., Weissman, J.S. *et al.* (2014) The coding and noncoding architecture of the *Caulobacter crescentus* genome. *PLoS Genet*, **10**, e1004463.
2. Thanbichler, M., Iniesta, A.A. and Shapiro, L. (2007) A comprehensive set of plasmids for vanillate- and xylose-inducible gene expression in *Caulobacter crescentus*. *Nucleic Acids Res*, **35**, e137.
3. Santos, P.M., Di Bartolo, I., Blatny, J.M., Zennaro, E. and Valla, S. (2001) New broad-host-range promoter probe vectors based on the plasmid RK2 replicon. *FEMS Microbiol Lett*, **195**, 91-96.
4. Larsen, R.A., Wilson, M.M., Guss, A.M. and Metcalf, W.W. (2002) Genetic analysis of pigment biosynthesis in *Xanthobacter autotrophicus* Py2 using a new, highly efficient transposon mutagenesis system that is functional in a wide variety of bacteria. *Arch Microbiol*, **178**, 193-201.
5. Corcoran, C.P., Podkaminski, D., Papenfort, K., Urban, J.H., Hinton, J.C. and Vogel, J. (2012) Superfolder GFP reporters validate diverse new mRNA targets of the classic porin regulator, MicF RNA. *Mol Microbiol*, **84**, 428-445.
6. Arellano, B.H., Ortiz, J.D., Manzano, J. and Chen, J.C. (2010) Identification of a dehydrogenase required for lactose metabolism in *Caulobacter crescentus*. *Appl Environ Microbiol*, **76**, 3004-3014.
7. Irnov, I., Wang, Z., Jannetty, N.D., Bustamante, J.A., Rhee, K.Y. and Jacobs-Wagner, C. (2017) Crosstalk between the tricarboxylic acid cycle and peptidoglycan synthesis in Caulobacter crescentus through the homeostatic control of alpha-ketoglutarate. *PLoS Genet*, **13**, e1006978.
